# Supplementary material for: Adoption of climate-resilient groundnut varieties increases agricultural production, consumption, and smallholder commercialization in West Africa
Source: Nat Commun. 2023 Aug 24;14:5175. doi: 10.1038/s41467-023-40781-1 (PMC10449883; doi:10.1038/s41467-023-40781-1)
Supplement: Supplementary file 1 — Supplementary information [file 41467_2023_40781_MOESM1_ESM.pdf]

Adoption of Climate-Resilient Groundnut Varieties Increases Agricultural Production, Consumption, and Smallholder Commercialization in West Africa

Supplementary Information

Martin Paul Jr. Tabe-Ojong, Jourdain Lokossou, Bisrat Gebrekidan, Hippolyte D. Affognon

Table of Contents

1 Supplementary Tables **4**
- 1.1 Supplementary Note 4
- 1.2 Descriptive statistics 6
- 1.3 Pooled OLS Regressions 8
- 1.4 Panel Regression 16
- 1.5 Cross country analysis 27

2 Robustness checks **32**
- 2.1 Control function approach 32
- 2.2 Hausman Taylor IV 35
- 2.3 Lewbels Test 37

3 Questionnaire **39**

List of Tables

S1 Descriptive statistics by year and adoption status . . . 7
S2 Full OLS estimates of the relationship between adoption and commercialization(Adoption) . 8
S3 Full OLS estimates of the relationship between adoption and commercialization (Area under Adoption) . . . 10
S4 Full OLS estimates of the relationship between adoption, production yields and consumption(Adoption) . . . 12
S5 OLS estimates of the relationship between adoption, production , yields and consumption(Area under Adoption) . . . 14
S6 Full 2SLS estimates of the relationship between adoption and commercialization . . . 16
S7 Full 2SLS estimates of the relationship between adoption (Area) and commercialization . . 18
S8 Full 2SLS estimates of the relationship between adoption, production , consumption and yields . . . 21
S9 Full 2SLS estimates of the relationship between continous adoption,quantity sold, consumption and yields . . . 24
S10 2SLS estimates of the relationship between adoption and commercialization and yield across countries . . . 28
S11 2SLS estimates of the relationship between adoption and commercialization, production and yield simultaneously . . . 31
S12 Control function estimations of the relationship between adoption and market participation 32
S13 HAUSMAN TAYLOR IV estimations . . . 35
S14 Lewbel instrumental variable estimations of the relationship between adoption and commercialization . . . 37

1 Supplementary Tables

1.1 Supplementary Note

We present the results of the estimation using the pooled FE-OLS model. Figure S1 presents the results of the relationship between the adoption of climate-resilient groundnut varieties and commercialization where we employ the linear probability model for binary outcomes. We present results when we consider adoption as a dummy and the extent of adoption of climate-resilient groundnut varieties. Considering adoption as a dummy, we establish a positive association with the commercialization outcomes; market participation, quantity of groundnut sold, and sales. Considering the area under adoption, we obtain negative estimates that are not statistically significant. However, this result could mean that increasing the area of cultivation of improved climate-resilient groundnut varieties is negatively correlated with market participation, quantity sold and the associated sales value. This negative relationship although not statistically significant could be due to diminishing returns when we consider the area under adoption. Otherwise, these negative results could be due to endogeneity issues which could lead to biased estimates. Given that we control for these endogeneity issues using the 2SLS and both household fixed effects and the correlated random effects model, we only use these results for comparison with the main estimation results.

Figure S1: OLS estimates of the relationship between adoption and commercialization

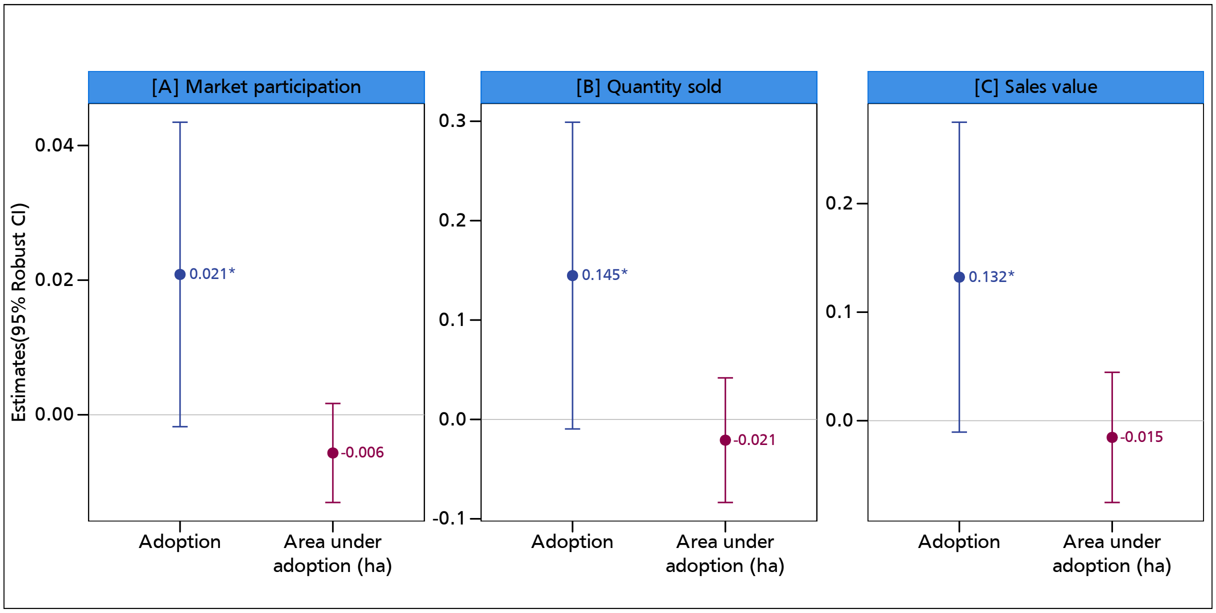

Note: The graph displays coefficients along with their corresponding 95% confidence intervals as error bars. The coefficents are estimated using OLS with n=8604 observation.The presence of an asterisk (*) above a coefficient indicates that the coefficient is statistically different from zero at a predetermined level of significance (*** p<0.01, ** p<0.05, * p<0.1).Statistical tests are two-sided t-tests. Full models are reported in S2 & S3 with Robust standard errors in parentheses and P-values in square brackets. Additional controls include age and educational level of the household head, dependency ratio, whether the household head is male, household size, cooperative membership, training, access to public and private extension, access to credits both in cash and kind, distance to nearest urban and village market, crop rotation, mixed cropping, labour, market price, input costs, area of cultivation, off-farm income and soil type.

Estimating the relationship between adoption of improved groundnuts, production, production value and land productivity using the FE-OLS model (Figure S2), we obtain positive coefficients for all outcomes. When we consider adoption as a dummy, we observe production and productivity increases of about 540Kg and 285Kg/ha respectively. Considering the scale of adoption, we observe that adoption of improved climate-smart groundnut varieties increases groundnut production by 240Kg and land productivity by approximately 60Kg/ha. The magnitudes here are positive indicating that adoption both when considered as a dummy as well as extent increases yield, production, and production value. The smaller magnitudes here might be indicative of diminishing returns as early highlighted. The positive and significant estimates of the area under adoption variable aligns with the tenets of the non-separable agricultural household model where the production, consumption and ultimately commercialization decisions of households are non-separable. This suggests that households would only participate in markets to the extent that the household food production and consumption needs are met.

Figure S2: OLS estimates of the relationship between adoption and commercialization

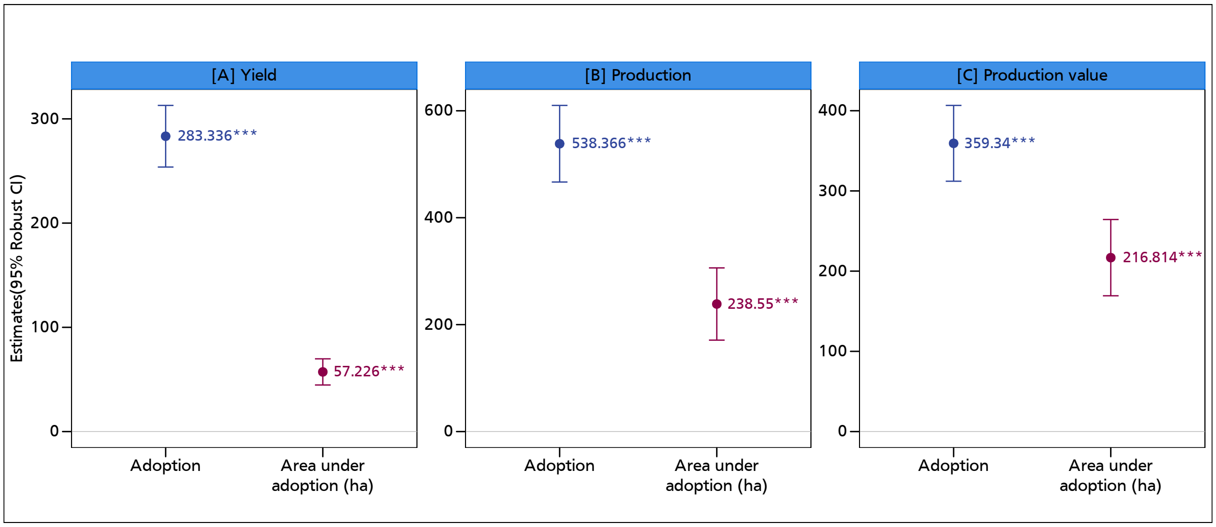

Note: The graph displays coefficients along with their corresponding 95% confidence intervals as error bars. The coefficents are estimated using OLS with n=8604 observation.The presence of an asterisk (*) above a coefficient indicates that the coefficient is statistically different from zero at a predetermined level of significance (*** p<0.01, ** p<0.05, * p<0.1). Statistical tests are two-sided t-tests. Full models are reported in S4 & S5 with Robust standard errors in parentheses and P-values in square brackets. Additional controls include age and educational level of the household head, dependency ratio, whether the household head is male, household size, cooperative membership, training, access to public and private extension, access to credits both in cash and kind, distance to nearest urban and village market, crop rotation, mixed cropping, labour, market price, input costs, area of cultivation, off-farm income and soil type.

1.2 Descriptive statistics

Table S1: Descriptive statistics by year and adoption status

	**2017**, N = 2868			**2018**, N = 2868			**2019**, N = 2868		
**Characteristic**	**Non-adopter**, N = 1,809	**Adopter**, N = 1,059	**p-value**	**Non-adopter**, N = 1,770	**Adopter**, N = 1,098	**p-value**	**Non-adopter**, N = 1,670	**Adopter**, N = 1,198	**p-value**
Country			<0.001			<0.001			<0.001
Ghana	327 (18%)	171 (16%)		353 (20%)	145 (13%)		340 (20%)	158 (13%)	
Mali	697 (39%)	143 (14%)		693 (39%)	147 (13%)		642 (38%)	198 (17%)	
Nigeria	785 (43%)	745 (70%)		724 (41%)	806 (73%)		688 (41%)	842 (70%)	
Age of household head (years)	48 (13)	47 (11)	0.073	49 (13)	47 (11)	<0.001	50 (12)	49 (12)	0.14
Sex of household head (dummy, male=1)	1,681 (93%)	1,004 (95%)	0.047	1,629 (92%)	1,056 (96%)	<0.001	1,546 (93%)	1,139 (95%)	0.007
Education level (Number of years)	2.5 (3.8)	3.4 (4.4)	<0.001	2.4 (3.8)	3.6 (4.4)	<0.001	2.1 (3.3)	3.9 (4.8)	<0.001
Household size (number of persons)	12 (7)	10 (6)	<0.001	12 (7)	10 (6)	<0.001	13 (10)	10 (7)	<0.001
Dependency ratio	1.59 (1.10)	1.77 (1.38)	0.029	1.64 (1.15)	1.69 (1.30)	0.8	1.74 (1.32)	1.95 (1.63)	0.015
Farmers group membership (dummy)	757 (42%)	551 (52%)	<0.001	771 (44%)	537 (49%)	0.005	696 (42%)	518 (43%)	0.4
Training on agriculture (dummy)	591 (33%)	473 (45%)	<0.001	557 (31%)	507 (46%)	<0.001	530 (32%)	565 (47%)	<0.001
Training on groundnut farming(dummy)	1,020 (56%)	587 (55%)	0.6	1,001 (57%)	606 (55%)	0.5	629 (38%)	766 (64%)	<0.001
Public agricultural extension service (number of visits)	1.21 (1.66)	3.32 (3.30)	<0.001	1.40 (1.84)	2.94 (3.29)	<0.001	1.71 (1.90)	2.47 (2.12)	<0.001
Private agricultural extension service (number of visits)	0.58 (0.90)	1.54 (1.89)	<0.001	0.62 (0.94)	1.44 (1.88)	<0.001	1.11 (1.33)	1.38 (1.57)	<0.001
Cash credit for groundnut farming (dummy)	32 (1.8%)	24 (2.3%)	0.4	30 (1.7%)	26 (2.4%)	0.2	49 (2.9%)	76 (6.3%)	<0.001
Credit in kind for groundnut farming (dummy)	62 (3.4%)	129 (12%)	<0.001	54 (3.1%)	137 (12%)	<0.001	87 (5.2%)	150 (13%)	<0.001
Distance to the nearest urban market (km)	15 (18)	11 (11)	<0.001	15 (19)	11 (11)	<0.001	13 (14)	12 (14)	<0.001
Distance the nearest village market (km)	3.8 (5.3)	3.5 (3.7)	0.004	3.9 (5.4)	3.4 (3.6)	0.003	4.8 (5.0)	3.6 (4.5)	<0.001
Crop rotation (dummy)	889 (49%)	397 (37%)	<0.001	905 (51%)	381 (35%)	<0.001	921 (55%)	393 (33%)	<0.001
Mixed Crops (dummy)	657 (36%)	448 (42%)	0.001	681 (38%)	424 (39%)	>0.9	725 (43%)	542 (45%)	0.3
Labor force (man.day)	3.9 (5.1)	6.5 (7.4)	<0.001	4.4 (5.5)	5.6 (7.1)	<0.001	7 (9)	7 (6)	<0.001
Unit selling price (USD/kg)	0.53 (0.07)	0.71 (0.08)	<0.001	0.53 (0.07)	0.72 (0.08)	<0.001	0.53 (0.07)	0.71 (0.09)	<0.001
Seed cost (USD/ha)	8 (16)	27 (19)	<0.001	8 (17)	25 (20)	<0.001	20 (21)	23 (19)	<0.001
Fertilizer cost (USD/ha)	17 (29)	53 (39)	<0.001	18 (30)	49 (40)	<0.001	19 (28)	49 (39)	<0.001
Pesticide cost (USD/ha)	4 (8)	14 (14)	<0.001	4 (8)	13 (14)	<0.001	6 (13)	11 (11)	<0.001
Labor cost (USD/ha)	21 (33)	49 (41)	<0.001	24 (34)	43 (41)	<0.001	50 (49)	50 (41)	0.031
Groundnut area (ha)	1.44 (1.47)	1.81 (1.62)	<0.001	1.49 (1.46)	1.72 (1.64)	<0.001	1.60 (1.47)	1.72 (1.32)	<0.001
Off-farm income (dummy)	80 (4.4%)	190 (18%)	<0.001	85 (4.8%)	185 (17%)	<0.001	142 (8.5%)	199 (17%)	<0.001
Clay soil (dummy)	279 (15%)	164 (15%)	>0.9	271 (15%)	172 (16%)	0.8	282 (17%)	207 (17%)	0.8
Sandy-clay soil (dummy)	987 (55%)	595 (56%)	0.4	977 (55%)	605 (55%)	>0.9	740 (44%)	516 (43%)	0.5
Silty soil (dummy)	281 (16%)	162 (15%)	0.9	278 (16%)	165 (15%)	0.6	306 (18%)	200 (17%)	0.3

[1] n (%); Mean (SD)

[2] Pearson's Chi-squared test; Wilcoxon rank sum test

Note: The table below presents a comparison between adopters and non-adopters over time. Two-sided t-tests were used for statistical testing, and the corresponding p-values are presented in the last column. The tests performed are Pearsons Chi-squared test for categorical variables and the Wilcoxon rank sum test for continuous variables.

1.3 Pooled OLS Regressions

Table S2: Full OLS estimates of the relationship between adoption and commercialization(Adoption)

	(1)	(2)	(3)
variables	Market participation	Quantity Sold	Sales value
Adoption dummy	0.021*	0.145*	0.132*
	(0.012)	(0.079)	(0.073)
	[0.071]	[0.066]	[0.069]
Age of household head (years)	-0.001**	-0.006**	-0.005***
	(0.000)	(0.002)	(0.002)
	[0.026]	[0.011]	[0.010]
Sex of household head (dummy, male=1)	-0.012	0.105	0.112
	(0.020)	(0.129)	(0.118)
	[0.554]	[0.416]	[0.341]
Education level (Number of years)	0.001	-0.005	-0.005
	(0.001)	(0.006)	(0.005)
	[0.530]	[0.393]	[0.308]
Household size (number of persons)	0.000	0.015***	0.015***
	(0.001)	(0.005)	(0.004)
	[0.642]	[0.001]	[0.001]
Farmers group membership (dummy)	0.022***	0.132***	0.120***
	(0.004)	(0.029)	(0.027)
	[0.000]	[0.000]	[0.000]
Training on agriculture (dummy)	-0.057***	-0.320***	-0.282***
	(0.011)	(0.074)	(0.068)
	[0.000]	[0.000]	[0.000]
Training on groundnut farming (dummy)	-0.021***	-0.154***	-0.143***
	(0.004)	(0.025)	(0.023)
	[0.000]	[0.000]	[0.000]
Public agricultural extension service (number of visits)	0.001	-0.017	-0.018
	(0.002)	(0.014)	(0.013)
	[0.644]	[0.222]	[0.168]
Private agricultural extension service (number of visits)	0.007**	0.034*	0.027
	(0.003)	(0.020)	(0.019)
	[0.022]	[0.092]	[0.159]
Cash credit for groundnut farming (dummy)	0.011	0.034	0.026
	(0.020)	(0.140)	(0.130)
	[0.591]	[0.806]	[0.842]
Credit in kind for groundnut farming (dummy)	-0.008	0.039	0.043
	(0.012)	(0.088)	(0.082)
	[0.520]	[0.654]	[0.604]
Distance to the nearest urban market (km)	-0.002***	-0.015***	-0.014***
	(0.000)	(0.002)	(0.002)
	[0.000]	[0.000]	[0.000]
Distance the nearest village market (km)	-0.004***	-0.021***	-0.019***
	(0.001)	(0.007)	(0.007)
	[0.000]	[0.004]	[0.005]
Crop rotation (dummy)	0.010	0.085	0.078
	(0.010)	(0.063)	(0.057)
	[0.313]	[0.177]	[0.173]
Mixed Crops (dummy)	0.003	-0.095*	-0.097**
	(0.008)	(0.051)	(0.047)
	[0.661]	[0.064]	[0.038]
Labor force (man.day)	0.002***	0.024***	0.023***
	(0.001)	(0.004)	(0.004)
	[0.000]	[0.000]	[0.000]
Unit selling price (USDkg)	0.068	0.579**	2.001***
	(0.042)	(0.286)	(0.264)
	[0.109]	[0.043]	[0.000]
Seed cost (USDha)	0.001***	0.010***	0.009***
	(0.000)	(0.002)	(0.002)
	[0.000]	[0.000]	[0.000]
Fertilizer cost (USDha)	0.000	0.001*	0.001*
	(0.000)	(0.001)	(0.001)
	[0.417]	[0.052]	[0.052]
Pesticide cost (USDha)	-0.000	0.003	0.003
	(0.000)	(0.002)	(0.002)

Table S2: Full OLS estimates of the relationship between adoption and commercialization(Adoption) *(continued)*

variables	Market participation	Quantity Sold	Sales value
	[0.160]	[0.212]	[0.129]
Labor cost (USDha)	0.000***	0.002***	0.002***
	(0.000)	(0.001)	(0.001)
	[0.000]	[0.002]	[0.002]
Groundnut area (ha)	0.019***	0.347***	0.335***
	(0.003)	(0.022)	(0.021)
	[0.000]	[0.000]	[0.000]
Off-farm income (dummy)	-0.033***	-0.151**	-0.135**
	(0.010)	(0.074)	(0.069)
	[0.002]	[0.040]	[0.049]
Dependency ratio	0.001	-0.002	-0.003
	(0.003)	(0.019)	(0.017)
	[0.820]	[0.894]	[0.869]
Clay soil (dummy)	-0.008	-0.097	-0.095
	(0.011)	(0.077)	(0.071)
	[0.463]	[0.212]	[0.182]
Sandy-clay soil (dummy)	0.007	0.035	0.031
	(0.009)	(0.061)	(0.056)
	[0.446]	[0.565]	[0.582]
Silty soil (dummy)	0.008	0.052	0.046
	(0.011)	(0.076)	(0.070)
	[0.471]	[0.494]	[0.516]
Observations	8,604	8,604	8,604
R-squared	0.274	0.421	0.451
F test	13.95	30.48	37.56

Note: The table presents the results of OLS regressions between adoption decision ('Adoption dummy') and market participation(1), quantity sold(2) and Sales value(3). with robust standard errors, where the standard errors are clustered. The statistical tests conducted are two-sided t-tests. P-values are denoted in square brackets. The presence of an asterisk (*) above a coefficient indicates that the coefficient is statistically different from zero at a predetermined level of significance (*** p<0.01, ** p<0.05, * p<0.1). All regressions include a comprehensive set of district fixed effects to control for potential unobserved heterogeneity.

Table S3: Full OLS estimates of the relationship between adoption and commercialization (Area under Adoption)

	(1)	(2)	(3)
variables	Market participation	Quantity Sold	Sales value
Area under adoption (ha)	-0.006	-0.021	-0.015
	(0.004)	(0.032)	(0.031)
	[0.130]	[0.514]	[0.615]
Age of household head (years)	-0.001**	-0.006**	-0.005***
	(0.000)	(0.002)	(0.002)
	[0.026]	[0.011]	[0.010]
Sex of household head (dummy, male=1)	-0.011	0.110	0.116
	(0.020)	(0.129)	(0.118)
	[0.580]	[0.396]	[0.325]
Education level (Number of years)	0.001	-0.005	-0.006
	(0.001)	(0.006)	(0.005)
	[0.555]	[0.386]	[0.305]
Household size (number of persons)	0.000	0.014***	0.014***
	(0.001)	(0.005)	(0.004)
	[0.710]	[0.002]	[0.001]
Farmers group membership (dummy)	0.022***	0.132***	0.120***
	(0.004)	(0.029)	(0.027)
	[0.000]	[0.000]	[0.000]
Training on agriculture (dummy)	-0.056***	-0.319***	-0.282***
	(0.011)	(0.074)	(0.068)
	[0.000]	[0.000]	[0.000]
Training on groundnut farming (dummy)	-0.021***	-0.155***	-0.144***
	(0.004)	(0.025)	(0.023)
	[0.000]	[0.000]	[0.000]
Public agricultural extension service (number of visits)	0.001	-0.015	-0.016
	(0.002)	(0.014)	(0.013)
	[0.523]	[0.290]	[0.222]
Private agricultural extension service (number of visits)	0.008***	0.042**	0.033*
	(0.003)	(0.020)	(0.019)
	[0.007]	[0.038]	[0.075]
Cash credit for groundnut farming (dummy)	0.011	0.038	0.030
	(0.020)	(0.140)	(0.131)
	[0.575]	[0.784]	[0.819]
Credit in kind for groundnut farming (dummy)	-0.006	0.052	0.053
	(0.012)	(0.087)	(0.082)
	[0.655]	[0.552]	[0.515]
Distance to the nearest urban market (km)	-0.002***	-0.015***	-0.014***
	(0.000)	(0.002)	(0.002)
	[0.000]	[0.000]	[0.000]
Distance the nearest village market (km)	-0.004***	-0.021***	-0.019***
	(0.001)	(0.007)	(0.007)
	[0.000]	[0.004]	[0.005]
Crop rotation (dummy)	0.009	0.080	0.074
	(0.010)	(0.063)	(0.058)
	[0.359]	[0.201]	[0.195]
Mixed Crops (dummy)	0.002	-0.100*	-0.102**
	(0.008)	(0.051)	(0.047)
	[0.742]	[0.051]	[0.030]
Labor force (man.day)	0.002***	0.023***	0.022***
	(0.001)	(0.004)	(0.004)
	[0.000]	[0.000]	[0.000]
Unit selling price (USDkg)	0.134***	0.982***	2.357***
	(0.034)	(0.235)	(0.218)
	[0.000]	[0.000]	[0.000]
Seed cost (USDha)	0.001***	0.010***	0.009***
	(0.000)	(0.002)	(0.002)
	[0.000]	[0.000]	[0.000]
Fertilizer cost (USDha)	0.000	0.002**	0.002**
	(0.000)	(0.001)	(0.001)
	[0.292]	[0.031]	[0.032]
Pesticide cost (USDha)	-0.000	0.004	0.004*
	(0.000)	(0.002)	(0.002)
	[0.264]	[0.139]	[0.083]

Table S3: Full OLS estimates of the relationship between adoption and commercialization (Area under Adoption) *(continued)*

variables	Market participation	Quantity Sold	Sales value
Labor cost (USDha)	0.000***	0.002***	0.002***
	(0.000)	(0.001)	(0.001)
	[0.000]	[0.002]	[0.002]
Groundnut area (ha)	0.022***	0.355***	0.342***
	(0.003)	(0.022)	(0.021)
	[0.000]	[0.000]	[0.000]
Off-farm income (dummy)	-0.033***	-0.150**	-0.134*
	(0.010)	(0.074)	(0.069)
	[0.002]	[0.041]	[0.051]
Dependency ratio	0.001	-0.003	-0.003
	(0.003)	(0.019)	(0.017)
	[0.839]	[0.885]	[0.863]
Clay soil (dummy)	-0.009	-0.101	-0.099
	(0.011)	(0.077)	(0.071)
	[0.408]	[0.189]	[0.163]
Sandy-clay soil (dummy)	0.006	0.032	0.028
	(0.009)	(0.060)	(0.055)
	[0.487]	[0.593]	[0.607]
Silty soil (dummy)	0.008	0.053	0.046
	(0.011)	(0.076)	(0.070)
	[0.468]	[0.489]	[0.510]
Observations	8,604	8,604	8,604
R-squared	0.273	0.421	0.451
F test	14.32	31.28	38.78

Note: The table presents the results of OLS regressions between area under adoption in ha ('Area under adoption') and market participation(1), quantity sold(2) and Sales value(3). Robust standard errors are in brackates. The statistical tests conducted are two-sided t-tests. P-values, denoted in square brackets. The presence of an asterisk (*) above a coefficient indicates that the coefficient is statistically different from zero at a predetermined level of significance (*** p<0.01, ** p<0.05, * p<0.1). All regressions include a comprehensive set of district fixed effects to control for potential unobserved heterogeneity.

Table S4: Full OLS estimates of the relationship between adoption, production yields and consumption(Adoption)

	(1)	(2)	(3)	(4)
variables	Production	Production value	Yield	Consumption
Adoption dummy	538.366***	359.340***	283.336***	48.891
	(36.536)	(24.099)	(15.075)	(40.829)
	0.000	[0.000]	[0.000]	[0.231]
Age of household head (years)	0.226	0.246	-0.023	2.086**
	(0.882)	(0.625)	(0.374)	(1.048)
	0.798	[0.694]	[0.952]	[0.047]
Sex of household head (dummy, male=1)	-17.450	-20.938	-24.355	-23.960
	(31.798)	(23.338)	(17.706)	(31.806)
	0.583	[0.370]	[0.169]	[0.451]
Education level (Number of years)	0.573	-0.462	1.681	11.339***
	(2.929)	(2.113)	(1.329)	(3.409)
	0.845	[0.827]	[0.206]	[0.001]
Household size (number of persons)	-2.087	-3.346**	0.531	-9.443***
	(1.918)	(1.312)	(0.633)	(2.320)
	0.277	[0.011]	[0.401]	[0.000]
Farmers group membership (dummy)	27.503*	23.696**	1.717	32.920**
	(14.436)	(10.143)	(5.305)	(16.310)
	0.057	[0.020]	[0.746]	[0.044]
Training on agriculture (dummy)	20.058	10.166	12.945	17.410
	(28.123)	(19.713)	(11.464)	(32.155)
	0.476	[0.606]	[0.259]	[0.588]
Training on groundnut farming (dummy)	-2.803	-3.008	-2.129	17.571
	(9.558)	(6.533)	(4.027)	(10.709)
	0.769	[0.645]	[0.597]	[0.101]
Public agricultural extension service (number of visits)	-10.965	-9.267*	-7.113**	21.836**
	(7.583)	(5.383)	(2.889)	(8.703)
	0.148	[0.085]	[0.014]	[0.012]
Private agricultural extension service (number of visits)	-20.005**	-13.667**	-3.053	-12.012
	(9.408)	(6.454)	(3.826)	(9.947)
	0.034	[0.034]	[0.425]	[0.227]
Cash credit for groundnut farming (dummy)	-116.583*	-96.283**	-3.990	-39.613
	(62.411)	(42.370)	(27.589)	(68.001)
	0.062	[0.023]	[0.885]	[0.560]
Credit in kind for groundnut farming (dummy)	19.798	39.725	-8.167	-40.616
	(52.308)	(37.817)	(19.779)	(54.772)
	0.705	[0.294]	[0.680]	[0.458]
Distance to the nearest urban market (km)	-0.254	-0.150	0.039	3.065***
	(0.828)	(0.553)	(0.353)	(0.900)
	0.759	[0.787]	[0.912]	[0.001]
Distance the nearest village market (km)	-3.361*	-2.097*	-1.617**	0.650
	(1.840)	(1.198)	(0.795)	(2.005)
	0.068	[0.080]	[0.042]	[0.746]
Crop rotation (dummy)	-54.106*	-50.565**	1.007	3.006
	(27.676)	(19.779)	(11.600)	(29.979)
	0.051	[0.011]	[0.931]	[0.920]
Mixed Crops (dummy)	40.551*	32.475**	-1.605	135.895***
	(21.514)	(15.265)	(9.425)	(22.840)
	0.059	[0.033]	[0.865]	[0.000]
Labor force (man.day)	-4.390	-3.713**	-1.619**	-8.452***
	(2.721)	(1.808)	(0.788)	(2.579)
	0.107	[0.040]	[0.040]	[0.001]
Unit selling price (USDkg)	36.726	1,208.590***	93.809*	60.809
	(128.183)	(90.647)	(55.544)	(141.705)
	0.774	[0.000]	[0.091]	[0.668]
Seed cost (USDha)	-0.542	-0.338	-0.371	-1.834***
	(0.569)	(0.402)	(0.257)	(0.615)
	0.341	[0.400]	[0.150]	[0.003]
Fertilizer cost (USDha)	-1.346***	-1.273***	-0.004	-2.452***
	(0.440)	(0.322)	(0.199)	(0.481)
	0.002	[0.000]	[0.983]	[0.000]
Pesticide cost (USDha)	2.260*	2.177**	0.024	-2.370**
	(1.289)	(0.999)	(0.531)	(1.139)
	0.080	[0.029]	[0.964]	[0.037]

Table S4: Full OLS estimates of the relationship between adoption, production yields and consumption(Adoption) *(continued)*

variables	Production	Production value	Yield	Consumption
Labor cost (USDha)	0.075	0.043	-0.082	-0.841***
	(0.265)	(0.191)	(0.131)	(0.244)
	0.777	[0.821]	[0.529]	[0.001]
Groundnut area (ha)	698.176***	436.072***	1.376	339.550***
	(20.072)	(13.993)	(3.300)	(24.390)
	0.000	[0.000]	[0.677]	[0.000]
Off-farm income (dummy)	-15.131	-9.056	-21.645	-70.211*
	(39.174)	(29.191)	(18.076)	(38.588)
	0.699	[0.756]	[0.231]	[0.069]
Dependency ratio	-10.922	-9.494*	-1.563	-3.289
	(7.625)	(5.439)	(3.538)	(8.883)
	0.152	[0.081]	[0.659]	[0.711]
Clay soil (dummy)	6.092	0.292	3.708	-32.831
	(29.406)	(20.300)	(13.795)	(33.239)
	0.836	[0.989]	[0.788]	[0.323]
Sandy-clay soil (dummy)	28.195	20.606	-1.381	-46.923
	(25.243)	(17.693)	(11.221)	(28.923)
	0.264	[0.244]	[0.902]	[0.105]
Silty soil (dummy)	20.218	14.880	12.882	-31.084
	(30.870)	(21.598)	(13.986)	(35.069)
	0.513	[0.491]	[0.357]	[0.375]
Observations	8,604	8,604	8,604	8,604
R-squared	0.616	0.594	0.181	0.248
F test	69.22	73.12	26.48	14.71

Note: The table presents the results of OLS regressions between area under adoption in ha ('Adoption dummy') and Production(1), production value(2) , Yield(3) and Consumption(4).Robust standard errors are in brackates. The statistical tests conducted are two-sided t-tests. P-values is denoted in square brackets. The presence of an asterisk (*) above a coefficient indicates that the coefficient is statistically different from zero at a predetermined level of significance (*** p<0.01, ** p<0.05, * p<0.1). All regressions include a comprehensive set of district fixed effects to control for potential unobserved heterogeneity.

Table S5: OLS estimates of the relationship between adoption, production , yields and consumption(Area under Adoption)

	(1)	(2)	(3)	(4)
variables	Production	Production value	Yield	Consumption
Area under adoption (ha)	238.550***	216.814***	57.226***	20.643
	(34.459)	(24.253)	(6.403)	(38.366)
	[0.000]	[0.000]	[0.000]	[0.591]
Age of household head (years)	0.377	0.374	0.025	2.099**
	(0.875)	(0.611)	(0.380)	(1.045)
	[0.667]	[0.541]	[0.948]	[0.045]
Sex of household head (dummy, male=1)	-17.307	-23.796	-20.776	-23.894
	(29.621)	(20.843)	(17.751)	(31.850)
	[0.559]	[0.254]	[0.242]	[0.453]
Education level (Number of years)	2.728	1.428	2.280*	11.527***
	(2.916)	(2.076)	(1.354)	(3.402)
	[0.350]	[0.492]	[0.092]	[0.001]
Household size (number of persons)	0.104	-1.326	1.023	-9.254***
	(1.852)	(1.243)	(0.641)	(2.271)
	[0.955]	[0.286]	[0.110]	[0.000]
Farmers group membership (dummy)	23.024	19.604*	0.668	32.533**
	(14.530)	(10.143)	(5.411)	(16.393)
	[0.113]	[0.053]	[0.902]	[0.047]
Training on agriculture (dummy)	19.017	8.794	13.199	17.327
	(28.199)	(19.422)	(11.653)	(32.241)
	[0.500]	[0.651]	[0.257]	[0.591]
Training on groundnut farming (dummy)	-6.485	-5.155	-4.435	17.231
	(9.625)	(6.519)	(4.090)	(10.707)
	[0.500]	[0.429]	[0.278]	[0.108]
Public agricultural extension service (number of visits)	-5.811	-6.431	-3.685	22.315**
	(7.401)	(5.123)	(2.924)	(8.721)
	[0.432]	[0.209]	[0.208]	[0.011]
Private agricultural extension service (number of visits)	-10.123	-10.434*	6.138	-11.055
	(9.116)	(6.215)	(3.799)	(9.781)
	[0.267]	[0.093]	[0.106]	[0.258]
Cash credit for groundnut farming (dummy)	-87.350	-74.251*	8.406	-37.003
	(63.173)	(42.902)	(28.328)	(67.864)
	[0.167]	[0.084]	[0.767]	[0.586]
Credit in kind for groundnut farming (dummy)	-8.897	6.830	-6.967	-42.978
	(50.662)	(35.263)	(20.115)	(53.109)
	[0.861]	[0.846]	[0.729]	[0.418]
Distance to the nearest urban market (km)	-0.735	-0.486	-0.196	3.022***
	(0.826)	(0.541)	(0.363)	(0.897)
	[0.374]	[0.369]	[0.590]	[0.001]
Distance the nearest village market (km)	-3.326*	-2.099*	-1.568**	0.653
	(1.806)	(1.139)	(0.794)	(2.005)
	[0.066]	[0.066]	[0.048]	[0.745]
Crop rotation (dummy)	-42.921	-38.056**	0.911	3.933
	(26.863)	(18.900)	(11.740)	(29.791)
	[0.110]	[0.044]	[0.938]	[0.895]
Mixed Crops (dummy)	34.291	30.585**	-7.613	135.286***
	(21.331)	(14.868)	(9.562)	(22.649)
	[0.108]	[0.040]	[0.426]	[0.000]
Labor force (man.day)	-4.084	-3.021	-2.037**	-8.433***
	(2.927)	(1.994)	(0.808)	(2.576)
	[0.163]	[0.130]	[0.012]	[0.001]
Unit selling price (USDkg)	512.083***	1,339.478***	565.109***	107.283
	(133.797)	(96.971)	(49.594)	(147.220)
	[0.000]	[0.000]	[0.000]	[0.466]
Seed cost (USDha)	0.128	0.059	0.042	-1.772***
	(0.546)	(0.370)	(0.259)	(0.607)
	[0.815]	[0.873]	[0.872]	[0.004]
Fertilizer cost (USDha)	-0.846**	-0.963***	0.288	-2.406***
	(0.431)	(0.312)	(0.201)	(0.473)
	[0.050]	[0.002]	[0.153]	[0.000]
Pesticide cost (USDha)	2.188*	1.732*	0.456	-2.369**
	(1.325)	(1.024)	(0.540)	(1.163)
	[0.099]	[0.091]	[0.398]	[0.042]

Table S5: OLS estimates of the relationship between adoption, production , yields and consumption(Area under Adoption) *(continued)*

variables	Production	Production value	Yield	Consumption
Labor cost (USDha)	0.010	-0.006	-0.110	-0.846***
	(0.257)	(0.179)	(0.133)	(0.245)
	[0.968]	[0.974]	[0.410]	[0.001]
Groundnut area (ha)	634.557***	376.027***	-11.249***	334.084***
	(20.141)	(13.418)	(3.596)	(25.656)
	[0.000]	[0.000]	[0.002]	[0.000]
Off-farm income (dummy)	-3.625	0.179	-17.434	-69.193*
	(38.674)	(28.513)	(18.365)	(38.584)
	[0.925]	[0.995]	[0.342]	[0.073]
Dependency ratio	-7.874	-6.761	-0.786	-3.025
	(7.614)	(5.353)	(3.592)	(8.851)
	[0.301]	[0.207]	[0.827]	[0.733]
Clay soil (dummy)	28.503	22.644	6.733	-30.927
	(29.032)	(19.653)	(14.049)	(32.761)
	[0.326]	[0.249]	[0.632]	[0.345]
Sandy-clay soil (dummy)	45.222*	36.851**	1.791	-45.463
	(24.917)	(17.077)	(11.460)	(28.606)
	[0.070]	[0.031]	[0.876]	[0.112]
Silty soil (dummy)	26.747	20.009	15.403	-30.505
	(30.838)	(21.038)	(14.258)	(34.996)
	[0.386]	[0.342]	[0.280]	[0.383]
Observations	8,604	8,604	8,604	8,604
R-squared	0.622	0.613	0.156	0.248
F test	64.51	71.32	16.55	14.65

Note: The table presents the results of OLS regressions between area under adoption in ha ('Area under adoption') and Production(1), production value(2) , Yield(3) and Consumption(4).Robust standard errors are in brackates. The statistical tests conducted are two-sided t-tests. P-values are denoted in square brackets. The presence of an asterisk (*) above a coefficient indicates that the coefficient is statistically different from zero at a predetermined level of significance (*** p<0.01, ** p<0.05, * p<0.1). All regressions include a comprehensive set of district fixed effects to control for potential unobserved heterogeneity.

1.4 Panel Regression

Table S6: Full 2SLS estimates of the relationship between adoption and commercialization

	(1)		(2)		(3)	
	Market participation		Quantity sold		Sales value	
variables	FE	RE	FE	RE	FE	RE
Adoption dummy	0.064***	0.053***	0.594***	0.544***	0.570***	0.526***
	(0.020)	(0.018)	(0.134)	(0.120)	(0.124)	(0.110)
	[0.001]	[0.002]	[0.000]	[0.000]	[0.000]	[0.000]
Age of household head (years)	0.002	-0.001*	-0.011	-0.006**	-0.013	-0.005**
	(0.003)	(0.000)	(0.024)	(0.003)	(0.022)	(0.003)
	[0.545]	[0.066]	[0.649]	[0.043]	[0.554]	[0.040]
Sex of household head (dummy, male=1)		-0.011		0.115		0.121
		(0.020)		(0.137)		(0.126)
		[0.572]		[0.401]		[0.335]
Education level (Number of years)		0.001		-0.005		-0.006
		(0.001)		(0.009)		(0.008)
		[0.676]		[0.563]		[0.487]
Household size (number of persons)	0.002***	0.001*	0.027***	0.020***	0.026***	0.019***
	(0.001)	(0.001)	(0.005)	(0.004)	(0.004)	(0.004)
	[0.004]	[0.082]	[0.000]	[0.000]	[0.000]	[0.000]
Farmers group membership (dummy)	0.022***	0.023***	0.124***	0.132***	0.111***	0.119***
	(0.005)	(0.004)	(0.035)	(0.028)	(0.032)	(0.026)
	[0.000]	[0.000]	[0.000]	[0.000]	[0.000]	[0.000]
Training on agriculture (dummy)	-0.043***	-0.052***	-0.314***	-0.318***	-0.287***	-0.285***
	(0.011)	(0.009)	(0.078)	(0.065)	(0.072)	(0.059)
	[0.000]	[0.000]	[0.000]	[0.000]	[0.000]	[0.000]
Training on groundnut farming (dummy)	-0.025***	-0.023***	-0.176***	-0.166***	-0.162***	-0.153***
	(0.003)	(0.003)	(0.023)	(0.021)	(0.021)	(0.019)
	[0.000]	[0.000]	[0.000]	[0.000]	[0.000]	[0.000]
Public agricultural extension service (number of visits)	0.002	0.002	-0.024	-0.020	-0.025	-0.021
	(0.002)	(0.002)	(0.016)	(0.014)	(0.015)	(0.013)
	[0.354]	[0.355]	[0.149]	[0.162]	[0.104]	[0.106]
Private agricultural extension service (number of visits)	0.003	0.004	0.045*	0.026	0.042*	0.021
	(0.003)	(0.003)	(0.024)	(0.020)	(0.022)	(0.019)
	[0.318]	[0.153]	[0.059]	[0.199]	[0.055]	[0.266]
Cash credit for groundnut farming (dummy)	-0.010	0.000	-0.186	-0.076	-0.193	-0.083
	(0.023)	(0.020)	(0.156)	(0.136)	(0.144)	(0.125)
	[0.663]	[0.995]	[0.234]	[0.573]	[0.180]	[0.505]
Credit in kind for groundnut farming (dummy)	-0.044***	-0.026*	-0.046	-0.019	-0.022	-0.006
	(0.016)	(0.014)	(0.109)	(0.094)	(0.100)	(0.086)
	[0.007]	[0.060]	[0.674]	[0.835]	[0.827]	[0.946]
Distance to the nearest urban market (km)	-0.000	-0.001***	-0.003	-0.009***	-0.003	-0.008***
	(0.000)	(0.000)	(0.002)	(0.002)	(0.002)	(0.002)
	[0.154]	[0.000]	[0.101]	[0.000]	[0.100]	[0.000]
Distance the nearest village market (km)	-0.003***	-0.003***	-0.013**	-0.017***	-0.011*	-0.015***
	(0.001)	(0.001)	(0.006)	(0.005)	(0.006)	(0.005)
	[0.003]	[0.000]	[0.046]	[0.002]	[0.056]	[0.003]
Crop rotation (dummy)	-0.023**	-0.005	-0.144**	-0.016	-0.135**	-0.014
	(0.011)	(0.009)	(0.072)	(0.061)	(0.066)	(0.056)
	[0.030]	[0.544]	[0.046]	[0.790]	[0.043]	[0.798]
Mixed Crops (dummy)	0.004	0.003	-0.063	-0.079	-0.066	-0.080*
	(0.009)	(0.007)	(0.061)	(0.050)	(0.056)	(0.046)
	[0.678]	[0.718]	[0.298]	[0.119]	[0.238]	[0.082]
Labor force (man.day)	0.002***	0.002***	0.027***	0.026***	0.026***	0.025***
	(0.001)	(0.001)	(0.005)	(0.004)	(0.004)	(0.004)
	[0.003]	[0.000]	[0.000]	[0.000]	[0.000]	[0.000]
Unit selling price (USDkg)	0.009	0.012	-0.134	-0.180	1.301***	1.249***
	(0.054)	(0.050)	(0.365)	(0.338)	(0.336)	(0.311)
	[0.873]	[0.803]	[0.714]	[0.596]	[0.000]	[0.000]
Seed cost (USDha)	0.002***	0.002***	0.011***	0.010***	0.010***	0.009***
	(0.000)	(0.000)	(0.002)	(0.001)	(0.001)	(0.001)
	[0.000]	[0.000]	[0.000]	[0.000]	[0.000]	[0.000]
Fertilizer cost (USDha)	0.000	0.000	0.001	0.001	0.001	0.001
	(0.000)	(0.000)	(0.001)	(0.001)	(0.001)	(0.001)
	[0.726]	[0.590]	[0.210]	[0.145]	[0.223]	[0.152]

Table S6: Full 2SLS estimates of the relationship between adoption and commercialization *(continued)*

variables	FE	RE	FE	RE	FE	RE
Pesticide cost (USDha)	-0.001***	-0.001**	-0.005	-0.002	-0.004	-0.001
	(0.000)	(0.000)	(0.003)	(0.003)	(0.003)	(0.002)
	[0.004]	[0.015]	[0.107]	[0.514]	[0.136]	[0.641]
Labor cost (USDha)	0.000***	0.000***	0.002***	0.002***	0.002***	0.002***
	(0.000)	(0.000)	(0.001)	(0.001)	(0.001)	(0.001)
	[0.001]	[0.000]	[0.004]	[0.001]	[0.004]	[0.001]
Groundnut area (ha)	0.003	0.013***	0.198***	0.282***	0.195***	0.274***
	(0.003)	(0.003)	(0.023)	(0.019)	(0.021)	(0.017)
	[0.410]	[0.000]	[0.000]	[0.000]	[0.000]	[0.000]
Off-farm income (dummy)	-0.020	-0.027**	-0.035	-0.101	-0.024	-0.088
	(0.014)	(0.012)	(0.098)	(0.083)	(0.090)	(0.076)
	[0.174]	[0.026]	[0.722]	[0.219]	[0.793]	[0.247]
Dependency ratio	0.002	0.001	0.013	0.003	0.012	0.003
	(0.003)	(0.003)	(0.022)	(0.018)	(0.020)	(0.016)
	[0.608]	[0.722]	[0.557]	[0.854]	[0.551]	[0.867]
Clay soil (dummy)	-0.016	-0.012	-0.161*	-0.120	-0.155*	-0.116*
	(0.013)	(0.011)	(0.087)	(0.073)	(0.080)	(0.068)
	[0.200]	[0.272]	[0.064]	[0.103]	[0.053]	[0.086]
Sandy-clay soil (dummy)	0.006	0.006	0.052	0.036	0.048	0.032
	(0.010)	(0.009)	(0.068)	(0.059)	(0.063)	(0.054)
	[0.525]	[0.485]	[0.448]	[0.541]	[0.449]	[0.552]
Silty soil (dummy)	-0.004	0.002	-0.032	0.010	-0.033	0.007
	(0.013)	(0.011)	(0.087)	(0.073)	(0.080)	(0.067)
	[0.732]	[0.864]	[0.708]	[0.893]	[0.676]	[0.922]
Constant	0.455**	0.987***	4.382***	7.194***	3.379***	5.831***
	(0.191)	(0.053)	(1.295)	(0.361)	(1.191)	(0.331)
	[0.017]	[0.000]	[0.001]	[0.000]	[0.005]	[0.000]
Observations	8,604	8,604	8,604	8,604	8,604	8,604
Number of id	2,868	2,868	2,868	2,868	2,868	2,868
District FE	YES	YES	YES	YES	YES	YES
Year FE	YES	YES	YES	YES	YES	YES
Standard errors in brackets						
*** p<0.01, ** p<0.05, * p<0.1						

Note: The table provides the results of 2SLS regressions examining the relationship between adoption decision ('Adoption dummy') and various factors related to Market participation (1), Quantity sold (2), and Sales value (3). The regressions were estimated using both Random Effect (RE) and Fixed Effect (FE) specifications, with robust standard errors shown in brackets. The statistical tests conducted were two-sided t-tests, and p-values are denoted in square brackets. Coefficients marked with an asterisk (*) indicate statistical significance at predetermined levels of significance (*** p<0.01, ** p<0.05, * p<0.1). To account for potential unobserved heterogeneity, all regressions include a comprehensive set of district fixed effects.

Table S7: Full 2SLS estimates of the relationship between adoption (Area) and commercialization

	(1)		(2)		(3)	
	Market participation		Quantity sold		Sales value	
variables	FE	RE	FE	RE	FE	RE
Area under adoption (ha)	0.044***	0.036***	0.414***	0.370***	0.397***	0.358***
	(0.014)	(0.013)	(0.094)	(0.086)	(0.087)	(0.079)
	[0.001]	[0.005]	[0.000]	[0.000]	[0.000]	[0.000]
Age of household head (years)	0.002	0.001	-0.015	-0.021	-0.017	-0.022
	(0.004)	(0.004)	(0.024)	(0.024)	(0.022)	(0.022)
	[0.639]	[0.792]	[0.529]	[0.390]	[0.440]	[0.316]
Sex of household head (dummy, male=1)		-0.015		0.065		0.073
		(0.020)		(0.140)		(0.128)
		[0.465]		[0.643]		[0.566]
Education level (Number of years)		0.001		-0.002		-0.002
		(0.001)		(0.009)		(0.008)
		[0.454]		[0.865]		[0.800]
Household size (number of persons)	0.002***	0.002***	0.030***	0.030***	0.029***	0.029***
	(0.001)	(0.001)	(0.005)	(0.005)	(0.005)	(0.005)
	[0.001]	[0.002]	[0.000]	[0.000]	[0.000]	[0.000]
Farmers group membership (dummy)	0.022***	0.022***	0.119***	0.120***	0.106***	0.107***
	(0.005)	(0.005)	(0.035)	(0.035)	(0.032)	(0.032)
	[0.000]	[0.000]	[0.001]	[0.001]	[0.001]	[0.001]
Training on agriculture (dummy)	-0.043***	-0.041***	-0.312***	-0.296***	-0.285***	-0.270***
	(0.012)	(0.012)	(0.078)	(0.079)	(0.072)	(0.072)
	[0.000]	[0.000]	[0.000]	[0.000]	[0.000]	[0.000]
Training on groundnut farming (dummy)	-0.025***	-0.025***	-0.178***	-0.181***	-0.165***	-0.167***
	(0.003)	(0.003)	(0.023)	(0.023)	(0.021)	(0.021)
	[0.000]	[0.000]	[0.000]	[0.000]	[0.000]	[0.000]
Public agricultural extension service (number of visits)	0.002	0.003	-0.025	-0.019	-0.026*	-0.021
	(0.002)	(0.002)	(0.017)	(0.017)	(0.015)	(0.015)
	[0.387]	[0.206]	[0.133]	[0.247]	[0.091]	[0.176]
Private agricultural extension service (number of visits)	0.004	0.003	0.046*	0.043*	0.043**	0.041*
	(0.003)	(0.003)	(0.024)	(0.024)	(0.022)	(0.022)
	[0.298]	[0.320]	[0.053]	[0.066]	[0.049]	[0.061]
Cash credit for groundnut farming (dummy)	-0.006	-0.005	-0.144	-0.139	-0.152	-0.148
	(0.023)	(0.023)	(0.157)	(0.157)	(0.145)	(0.145)
	[0.811]	[0.845]	[0.359]	[0.376]	[0.293]	[0.307]
Credit in kind for groundnut farming (dummy)	-0.048***	-0.047***	-0.090	-0.080	-0.064	-0.056
	(0.016)	(0.016)	(0.111)	(0.111)	(0.102)	(0.103)
	[0.003]	[0.004]	[0.418]	[0.470]	[0.529]	[0.587]
Distance to the nearest urban market (km)	-0.000	-0.001***	-0.003	-0.009***	-0.003	-0.008***
	(0.000)	(0.000)	(0.002)	(0.002)	(0.002)	(0.002)
	[0.182]	[0.000]	[0.131]	[0.000]	[0.131]	[0.000]
Distance the nearest village market (km)	-0.003***	-0.003***	-0.012*	-0.016***	-0.010*	-0.015***
	(0.001)	(0.001)	(0.006)	(0.005)	(0.006)	(0.005)
	[0.004]	[0.000]	[0.065]	[0.002]	[0.078]	[0.003]
Crop rotation (dummy)	-0.020*	-0.019*	-0.114	-0.101	-0.106	-0.093

Table S7: Full 2SLS estimates of the relationship between adoption (Area) and commercialization *(continued)*

variables	FE	RE	FE	RE	FE	RE
	(0.011)	(0.011)	(0.074)	(0.074)	(0.068)	(0.068)
	[0.068]	[0.080]	[0.123]	[0.171]	[0.122]	[0.170]
Mixed Crops (dummy)	0.005	0.002	-0.049	-0.068	-0.052	-0.069
	(0.009)	(0.009)	(0.061)	(0.061)	(0.056)	(0.056)
	[0.563]	[0.837]	[0.422]	[0.268]	[0.352]	[0.219]
Labor force (man.day)	0.002***	0.002***	0.028***	0.028***	0.027***	0.027***
	(0.001)	(0.001)	(0.005)	(0.005)	(0.004)	(0.004)
	[0.002]	[0.002]	[0.000]	[0.000]	[0.000]	[0.000]
Unit selling price (USDkg)	0.017	0.037	-0.055	0.045	1.376***	1.469***
	(0.052)	(0.050)	(0.355)	(0.337)	(0.326)	(0.310)
	[0.744]	[0.449]	[0.876]	[0.895]	[0.000]	[0.000]
Seed cost (USDha)	0.002***	0.002***	0.011***	0.011***	0.010***	0.010***
	(0.000)	(0.000)	(0.002)	(0.002)	(0.001)	(0.001)
	[0.000]	[0.000]	[0.000]	[0.000]	[0.000]	[0.000]
Fertilizer cost (USDha)	0.000	0.000	0.002*	0.002*	0.002*	0.002*
	(0.000)	(0.000)	(0.001)	(0.001)	(0.001)	(0.001)
	[0.437]	[0.365]	[0.067]	[0.061]	[0.068]	[0.063]
Pesticide cost (USDha)	-0.001***	-0.001***	-0.006**	-0.007**	-0.006**	-0.006**
	(0.000)	(0.000)	(0.003)	(0.003)	(0.003)	(0.003)
	[0.002]	[0.001]	[0.038]	[0.018]	[0.047]	[0.023]
Labor cost (USDha)	0.000***	0.000***	0.002***	0.003***	0.002***	0.002***
	(0.000)	(0.000)	(0.001)	(0.001)	(0.001)	(0.001)
	[0.000]	[0.000]	[0.002]	[0.001]	[0.002]	[0.001]
Groundnut area (ha)	-0.009*	-0.006	0.085**	0.103***	0.087***	0.103***
	(0.005)	(0.005)	(0.035)	(0.034)	(0.033)	(0.031)
	[0.073]	[0.221]	[0.016]	[0.002]	[0.008]	[0.001]
Off-farm income (dummy)	-0.019	-0.021	-0.031	-0.043	-0.020	-0.031
	(0.015)	(0.015)	(0.099)	(0.099)	(0.091)	(0.091)
	[0.186]	[0.143]	[0.755]	[0.661]	[0.827]	[0.730]
Dependency ratio	0.002	0.002	0.017	0.020	0.016	0.019
	(0.003)	(0.003)	(0.022)	(0.022)	(0.020)	(0.020)
	[0.509]	[0.457]	[0.430]	[0.365]	[0.420]	[0.355]
Clay soil (dummy)	-0.015	-0.010	-0.143	-0.102	-0.138*	-0.098
	(0.013)	(0.011)	(0.088)	(0.074)	(0.081)	(0.068)
	[0.260]	[0.345]	[0.103]	[0.171]	[0.088]	[0.151]
Sandy-clay soil (dummy)	0.008	0.008	0.068	0.060	0.063	0.056
	(0.010)	(0.009)	(0.069)	(0.059)	(0.064)	(0.055)
	[0.422]	[0.354]	[0.324]	[0.310]	[0.320]	[0.305]
Silty soil (dummy)	-0.005	0.002	-0.034	0.012	-0.035	0.009
	(0.013)	(0.011)	(0.087)	(0.074)	(0.080)	(0.068)
	[0.723]	[0.882]	[0.696]	[0.872]	[0.665]	[0.894]
Constant	0.817***	1.032***	7.214***	7.482***	5.972***	6.123***
	(0.183)	(0.078)	(1.248)	(0.534)	(1.148)	(0.490)
	[0.000]	[0.000]	[0.000]	[0.000]	[0.000]	[0.000]
Observations	8,604	8,604	8,604	8,604	8,604	8,604
Number of id	2,868	2,868	2,868	2,868	2,868	2,868

Table S7: Full 2SLS estimates of the relationship between adoption (Area) and commercialization *(continued)*

variables	FE	RE	FE	RE	FE	RE
District FE	YES	YES	YES	YES	YES	YES
Year FE	YES	YES	YES	YES	YES	YES
Standard errors in brackets						
*** p<0.01, ** p<0.05, * p<0.1						

Note: The table provides the results of 2SLS regressions examining the relationship between area of adoption in ha ('Area under adoption') and various factors related to Market participation (1), Quantity sold (2), and Sales value (3). The regressions were estimated using both Random Effect (RE) and Fixed Effect (FE) specifications, with robust standard errors shown in brackets. The statistical tests conducted were two-sided t-tests, and p-values are denoted in square brackets. Coefficients marked with an asterisk (*) indicate statistical significance at predetermined levels of significance (*** p<0.01, ** p<0.05, * p<0.1). To account for potential unobserved heterogeneity, all regressions include a comprehensive set of district fixed effects.

Table S8: Full 2SLS estimates of the relationship between adoption, production , consumption and yields

	(1)			(2)		
variables	Yield	Production value	Consumption	Yield	Production value	Consumption
Adoption dummy	344.491***	476.159***	213.525***			
	(31.119)	(48.846)	(73.166)			
	[0.000]	[0.000]	[0.004]			
Area under adoption				240.044***	331.791***	148.786***
				(22.765)	(33.348)	(51.027)
				[0.000]	[0.000]	0.004
Age of household head	-1.764	-0.649	24.046*	-4.263	-4.102	22.497*
	(5.487)	(8.612)	(12.900)	(5.779)	(8.466)	(12.954)
	[0.748]	[0.940]	[0.062]	[0.461]	[0.628]	0.082
Household size	1.631	-1.605	-9.816***	3.611***	1.133	-8.588***
	(1.119)	(1.756)	(2.630)	(1.193)	(1.747)	(2.674)
	[0.145]	[0.361]	[0.000]	[0.002]	[0.517]	0.001
Farmers group membership	-4.339	17.512	36.037*	-7.206	13.551	34.260*
	(7.999)	(12.556)	(18.808)	(8.413)	(12.324)	(18.857)
	[0.587]	[0.163]	[0.055]	[0.392]	[0.272]	0.069
Training on agriculture	27.801	12.633	63.381	29.038	14.343	64.148
	(18.021)	(28.286)	(42.370)	(18.916)	(27.710)	(42.400)
	[0.123]	[0.655]	[0.135]	[0.125]	[0.605]	0.130
Training on groundnut farming	1.255	6.025	48.752***	-0.402	3.735	47.725***
	(5.354)	(8.404)	(12.588)	(5.612)	(8.221)	(12.579)
	[0.815]	[0.473]	[0.000]	[0.943]	[0.650]	0.000
Public agricultural extension service	-2.771	-4.887	31.133***	-3.482	-5.869	30.692***
	(3.800)	(5.964)	(8.934)	(3.995)	(5.853)	(8.956)
	[0.466]	[0.413]	[0.000]	[0.383]	[0.316]	0.001
Private agricultural extension service	-4.178	-11.633	-25.896**	-3.336	-10.469	-25.374**
	(5.470)	(8.586)	(12.862)	(5.728)	(8.391)	(12.839)
	[0.445]	[0.175]	[0.044]	[0.560]	[0.212]	0.048
Cash credit for groundnut farming	-34.959	-100.824*	68.337	-10.665	-67.245	83.395
	(36.180)	(56.789)	(85.065)	(37.932)	(55.566)	(85.023)
	[0.334]	[0.076]	[0.422]	[0.779]	[0.226]	0.327
Credit in kind for groundnut farming	-11.792	32.827	-16.957	-37.424	-2.602	-32.844
	(25.272)	(39.667)	(59.418)	(26.818)	(39.286)	(60.113)
	[0.641]	[0.408]	[0.775]	[0.163]	[0.947]	0.585
Distance to the nearest urban market	0.277	-0.795	1.917*	0.406	-0.616	1.997*
	(0.458)	(0.720)	(1.078)	(0.482)	(0.706)	(1.081)
	[0.546]	[0.270]	[0.075]	[0.400]	[0.383]	0.065
Distance the nearest village market	-0.900	-3.282	-1.691	-0.410	-2.604	-1.387
	(1.483)	(2.328)	(3.487)	(1.556)	(2.280)	(3.488)
	[0.544]	[0.159]	[0.628]	[0.792]	[0.253]	0.691
Crop rotation	0.657	-57.944**	95.764**	18.209	-33.684	106.643***
	(16.734)	(26.266)	(39.344)	(17.858)	(26.160)	(40.029)
	[0.969]	[0.027]	[0.015]	[0.308]	[0.198]	0.008
Mixed Crops	-3.981	-0.791	121.324***	4.068	10.334	126.313***
	(14.033)	(22.026)	(32.993)	(14.765)	(21.630)	(33.096)

Table S8: Full 2SLS estimates of the relationship between adoption, production , consumption and yields *(continued)*

variables	Yield	Production value	Consumption	Yield	Production value	Consumption
	[0.777]	[0.971]	[0.000]	[0.783]	[0.633]	0.000
Labor force	-1.834*	-3.726**	-6.969***	-0.927	-2.472	-6.407**
	(1.086)	(1.705)	(2.553)	(1.151)	(1.685)	(2.579)
	[0.091]	[0.029]	[0.006]	[0.420]	[0.142]	0.013
Unit selling price	-4.303	944.429***	-291.865	41.071	1,007.144***	-263.742
	(84.567)	(132.740)	(198.831)	(85.482)	(125.222)	(191.606)
	[0.959]	[0.000]	[0.142]	[0.631]	[0.000]	0.169
Seed cost	-0.316	-0.706	-3.200***	0.084	-0.152	-2.952***
	(0.362)	(0.569)	(0.852)	(0.377)	(0.552)	(0.845)
	[0.382]	[0.215]	[0.000]	[0.823]	[0.783]	0.000
Fertilizer cost	-0.326	-1.623***	-4.888***	0.038	-1.120***	-4.662***
	(0.248)	(0.389)	(0.583)	(0.259)	(0.380)	(0.581)
	[0.188]	[0.000]	[0.000]	[0.884]	[0.003]	0.000
Pesticide cost	0.479	2.361**	0.046	-0.476	1.042	-0.545
	(0.683)	(1.072)	(1.606)	(0.743)	(1.088)	(1.665)
	[0.483]	[0.028]	[0.977]	[0.522]	[0.338]	0.743
Labor cost	-0.132	-0.216	-0.937**	0.017	-0.010	-0.845**
	(0.180)	(0.282)	(0.423)	(0.190)	(0.278)	(0.426)
	[0.464]	[0.444]	[0.027]	[0.929]	[0.970]	0.047
Groundnut area	4.688	442.704***	362.297***	-60.607***	352.452***	321.825***
	(5.273)	(8.277)	(12.398)	(8.544)	(12.516)	(19.151)
	[0.374]	[0.000]	[0.000]	[0.000]	[0.000]	0.000
Off-farm income	-32.969	-30.255	-96.324*	-30.662	-27.066	-94.894*
	(22.728)	(35.674)	(53.437)	(23.859)	(34.951)	(53.479)
	[0.147]	[0.396]	[0.071]	[0.199]	[0.439]	0.076
Dependency ratio	-2.550	-8.979	-1.541	0.101	-5.315	0.102
	(5.046)	(7.920)	(11.863)	(5.310)	(7.778)	(11.902)
	[0.613]	[0.257]	[0.897]	[0.985]	[0.494]	0.993
Clay soil	-8.783	-4.912	-89.820*	1.570	9.397	-83.404*
	(20.148)	(31.625)	(47.372)	(21.198)	(31.052)	(47.514)
	[0.663]	[0.877]	[0.058]	[0.941]	[0.762]	0.079
Sandy-clay soil	-6.730	23.207	-70.619*	2.638	36.156	-64.812*
	(15.832)	(24.851)	(37.225)	(16.644)	(24.382)	(37.307)
	[0.671]	[0.350]	[0.058]	[0.874]	[0.138]	0.082
Silty soil	18.191	38.862	-15.868	17.234	37.540	-16.460
	(20.049)	(31.470)	(47.139)	(21.050)	(30.837)	(47.184)
	[0.364]	[0.217]	[0.736]	[0.413]	[0.223]	0.727
Constant	578.046**	-644.945	-1,294.580*	743.725**	-415.941	-1,191.887*
	(288.035)	(452.114)	(677.222)	(305.495)	(447.516)	(684.760)
	[0.045]	[0.154]	[0.056]	[0.015]	[0.353]	0.082
Observations	8,604	8,604	8,604	8,604	8,604	8,604
Number of id	2,868	2,868	2,868	2,868	2,868	2,868
District FE	YES	YES	YES	YES	YES	YES
Year FE	YES	YES	YES	YES	YES	YES

Standard errors in brackets

*** p<0.01, ** p<0.05, * p<0.1

Table S8: Full 2SLS estimates of the relationship between adoption, production , consumption and yields *(continued)*

variables	Yield	Production value	Consumption	Yield	Production value	Consumption

Note: The table provides the results of 2SLS regressions examining the relationship between adoption decision ('Adoption dummy') and area of adoption in ha ('Area under adoption') and various factors related to Yield, production and Consumption. The regressions were estimated using Fixed Effect (FE) specifications, with robust standard errors shown in brackets. The statistical tests conducted were two-sided t-tests, and p-values are denoted in square brackets. Coefficients marked with an asterisk (*) indicate statistical significance at predetermined levels of significance (*** p<0.01, ** p<0.05, * p<0.1). To account for potential unobserved heterogeneity, all regressions include a comprehensive set of district fixed effects.

Table S9: Full 2SLS estimates of the relationship between continous adoption,quantity sold, consumption and yields

	(1)	(2)	(3)
variable	Yield	Consumption	Quantity sold
Three years continuous adoption	1,241.558***	442.096*	3.459***
	(109.207)	(232.261)	(0.843)
	[0.000]	[0.057]	[0.000]
Age of household head	3.043	29.136**	-0.010
	(6.770)	(12.811)	(0.025)
	[0.653]	[0.023]	[0.696]
Sex of household head	-5.704	2.115	0.100
	(26.148)	(53.072)	(0.148)
	[0.827]	[0.968]	[0.498]
Education level	1.786	11.093***	-0.005
	(1.659)	(3.367)	(0.009)
	[0.282]	[0.001]	[0.629]
Household size	1.422	-9.907***	0.026***
	(1.386)	(2.621)	(0.005)
	[0.305]	[0.000]	[0.000]
Farmers group membership	-1.115	35.620*	0.130***
	(9.920)	(18.766)	(0.037)
	[0.910]	[0.058]	[0.000]
Training on agriculture	34.339	64.568	-0.291***
	(22.390)	(42.357)	(0.083)
	[0.125]	[0.127]	[0.000]
Training on groundnut farming	-2.045	46.483***	-0.184***
	(6.615)	(12.517)	(0.025)
	[0.757]	[0.000]	[0.000]
Public agricultural extension service	1.450	33.164***	-0.012
	(4.706)	(8.902)	(0.017)
	[0.758]	[0.000]	[0.484]
Private agricultural extension service	9.044	-18.473	0.062**
	(6.622)	(12.533)	(0.025)
	[0.172]	[0.141]	[0.012]
Cash credit for groundnut farming	-1.744	94.178	-0.128
	(44.669)	(84.507)	(0.165)
	[0.969]	[0.265]	[0.438]
Credit in kind for groundnut farming	13.682	-1.986	-0.004
	(31.369)	(59.343)	(0.116)
	[0.663]	[0.973]	[0.970]
Distance to the nearest urban market	0.111	3.014***	-0.009***
	(0.490)	(0.944)	(0.002)
	[0.821]	[0.001]	[0.000]
Distance the nearest village market	-3.430***	-0.960	-0.019***
	(1.319)	(2.580)	(0.006)
	[0.009]	[0.710]	[0.001]
Crop rotation	-28.885	71.025*	-0.170**
	(20.471)	(38.744)	(0.076)

Table S9: Full 2SLS estimates of the relationship between continous adoption,quantity sold, consumption and yields *(continued)*

variable	Yield	Consumption	Quantity sold
	[0.158]	[0.067]	[0.025]
Mixed Crops	-9.799	120.908***	-0.089
	(17.415)	(32.947)	(0.064)
	[0.574]	[0.000]	[0.170]
Labor force	-3.194**	-7.905***	0.025***
	(1.345)	(2.545)	(0.005)
	[0.018]	[0.002]	[0.000]
Unit selling price	716.738***	151.924	1.093***
	(66.187)	(125.209)	(0.245)
	[0.000]	[0.225]	[0.000]
Seed cost	0.142	-2.860***	0.011***
	(0.446)	(0.843)	(0.002)
	[0.750]	[0.001]	[0.000]
Fertilizer cost	-0.087	-4.674***	0.002
	(0.306)	(0.579)	(0.001)
	[0.777]	[0.000]	[0.105]
Pesticide cost	2.303***	1.387	-0.003
	(0.825)	(1.561)	(0.003)
	[0.005]	[0.374]	[0.344]
Labor cost	-0.120	-1.002**	0.002***
	(0.222)	(0.420)	(0.001)
	[0.589]	[0.017]	[0.005]
Groundnut area	8.132	362.101***	0.209***
	(6.546)	(12.384)	(0.024)
	[0.214]	[0.000]	[0.000]
Off-farm income	-29.912	-90.212*	-0.041
	(28.166)	(53.284)	(0.104)
	[0.288]	[0.090]	[0.697]
Dependency ratio	-4.058	-3.783	0.013
	(6.265)	(11.852)	(0.023)
	[0.517]	[0.750]	[0.566]
Clay soil	-8.107	-38.002	-0.149*
	(17.912)	(35.025)	(0.078)
	[0.651]	[0.278]	[0.057]
Sandy-clay soil	4.858	-41.520	0.053
	(14.477)	(28.260)	(0.062)
	[0.737]	[0.142]	[0.392]
Silty soil	7.944	-28.525	0.004
	(17.914)	(35.019)	(0.078)
	[0.657]	[0.415]	[0.963]
Constant	2,586.162***	434.444	12.715***
	(214.256)	(451.470)	(1.574)
	[0.000]	[0.336]	[0.000]
Observations	8,604	8,604	8,604
Number of id	2,868	2,868	2,868
District FE	YES	YES	YES

Table S9: Full 2SLS estimates of the relationship between continous adoption,quantity sold, consumption and yields *(continued)*

variable	Yield	Consumption	Quantity sold
Year FE	YES	YES	YES
Standard errors in brackets			
*** p<0.01, ** p<0.05, * p<0.1			

Note: The table presents the results of 2SLS regressions, which were conducted to examine the relationship between continuous adoption (Three years) and various factors associated with Yield (1), Consumption (2), and quantity sold (3). The regressions employed Random Effect (RE) specifications, and robust standard errors are reported in brackets. Two-sided t-tests were employed for the statistical tests, and p-values are indicated within square brackets. Coefficients marked with an asterisk (*) signify statistical significance at predetermined levels of significance (*** p<0.01, ** p<0.05, * p<0.1). To account for potential unobserved heterogeneity, all regressions include a comprehensive set of district fixed effects.

1.5 Cross country analysis

Table S10: 2SLS estimates of the relationship between adoption and commercialization and yield across countries

	Ghana			Mali			Nigeria		
variables	Yield	Consumption	Sales value	Yield	Consumption	Sales value	Yield	Consumption	Sales value
Adoption dummy	379.921***	154.917	0.934	36.417	3.920	0.156	376.238***	326.944***	0.047***
	(147.299)	(386.300)	(0.999)	(67.499)	(276.291)	(0.720)	(25.809)	(77.817)	(0.011)
	[0.010]	[0.688]	[0.350]	[0.590]	[0.989]	[0.829]	[0.000]	[0.000]	[0.000]
Age of household head (years)	10.835	11.925	0.009	2.087	-27.131	0.305**	-3.271	37.415*	-0.004
	(17.731)	(37.728)	(0.098)	(15.271)	(46.140)	(0.120)	(8.938)	(20.347)	(0.003)
	[0.541]	[0.752]	[0.926]	[0.891]	[0.557]	[0.011]	[0.714]	[0.066]	[0.210]
Sex of household head (dummy, male=1)	-61.230**			40.096			20.564		
	(29.154)			(27.632)			(48.953)		
	[0.036]			[0.147]			[0.674]		
Education level (Number of years)	0.036			-2.163			3.166*		
	(2.877)			(2.391)			(1.747)		
	[0.990]			[0.366]			[0.070]		
Household size (number of persons)	0.585	-3.491	0.024	1.976*	-0.273	0.006	0.016	-23.908***	0.003***
	(4.547)	(8.785)	(0.023)	(1.032)	(3.145)	(0.008)	(2.300)	(5.228)	(0.001)
	[0.898]	[0.691]	[0.284]	[0.056]	[0.931]	[0.460]	[0.995]	[0.000]	[0.000]
Farmers group membership (dummy)	6.302	-14.137	0.539***	-3.090	-35.600	0.010	-1.294	78.951**	0.008*
	(17.974)	(35.238)	(0.091)	(10.207)	(30.863)	(0.080)	(14.137)	(32.153)	(0.004)
	[0.726]	[0.688]	[0.000]	[0.762]	[0.249]	[0.905]	[0.927]	[0.014]	[0.060]
Training on agriculture (dummy)	41.600	-37.802	0.115	29.363	71.667	-0.558***	25.704	6.443	-0.005
	(47.250)	(91.163)	(0.236)	(19.662)	(59.561)	(0.155)	(32.492)	(73.830)	(0.010)
	[0.379]	[0.678]	[0.627]	[0.135]	[0.229]	[0.000]	[0.429]	[0.930]	[0.599]
Training on groundnut farming (dummy)	6.240	-24.021	-0.064	1.657	13.627	-0.374***	-2.704	104.530***	0.003
	(15.577)	(29.981)	(0.078)	(10.355)	(31.243)	(0.081)	(11.289)	(25.648)	(0.004)
	[0.689]	[0.423]	[0.411]	[0.873]	[0.663]	[0.000]	[0.811]	[0.000]	[0.472]
Public agricultural extension service (number of visits)	0.476	-52.543***	-0.007	0.818	7.267	-0.009	-0.223	83.734***	0.001
	(7.916)	(15.281)	(0.040)	(6.670)	(20.873)	(0.054)	(6.118)	(13.874)	(0.002)
	[0.952]	[0.001]	[0.863]	[0.902]	[0.728]	[0.867]	[0.971]	[0.000]	[0.458]
Private agricultural extension service (number of visits)	-4.263	-34.192	0.044	10.796	33.269	0.053	-0.868	-36.540	0.002
	(10.725)	(21.666)	(0.056)	(7.477)	(23.968)	(0.062)	(11.114)	(25.397)	(0.003)
	[0.691]	[0.115]	[0.433]	[0.149]	[0.165]	[0.395]	[0.938]	[0.150]	[0.519]
Cash credit for groundnut farming (dummy)	119.032	190.479	-0.183	49.252	213.972	-0.646*	-96.654	-70.031	0.034*
	(92.150)	(179.190)	(0.463)	(43.001)	(133.006)	(0.347)	(59.790)	(136.902)	(0.019)
	[0.196]	[0.288]	[0.693]	[0.252]	[0.108]	[0.063]	[0.106]	[0.609]	[0.074]
Credit in kind for groundnut farming (dummy)	-57.439	51.755	-1.611***	48.422	109.702	0.249	-30.079	-58.335	0.074***
	(64.879)	(126.936)	(0.328)	(40.497)	(122.310)	(0.319)	(37.306)	(84.634)	(0.012)
	[0.376]	[0.683]	[0.000]	[0.232]	[0.370]	[0.435]	[0.420]	[0.491]	[0.000]
Distance to the nearest urban market (km)	2.750	4.297	0.113***	-0.081	3.583	-0.023***	-0.041	0.658	-0.000**
	(2.519)	(7.631)	(0.020)	(0.539)	(2.727)	(0.007)	(0.549)	(1.340)	(0.000)
	[0.275]	[0.573]	[0.000]	[0.880]	[0.189]	[0.001]	[0.940]	[0.624]	[0.031]
Distance the nearest village market (km)	-0.371	6.343	-0.013	-2.758***	-2.965	-0.018*	0.564	-6.280	0.003**
	(3.150)	(8.867)	(0.023)	(0.884)	(3.944)	(0.010)	(2.840)	(9.324)	(0.001)
	[0.906]	[0.474]	[0.575]	[0.002]	[0.452]	[0.084]	[0.843]	[0.501]	[0.040]
Crop rotation (dummy)	52.243	189.254**	-0.259	-19.076	-102.821	0.593***	-8.740	101.439	-0.009
	(38.965)	(79.682)	(0.206)	(24.486)	(75.257)	(0.196)	(26.837)	(61.802)	(0.008)

Table S10: 2SLS estimates of the relationship between adoption and commercialization and yield across countries *(continued)*

variables	Yield	Consumption	Sales value	Yield	Consumption	Sales value	Yield	Consumption	Sales value
	[0.180]	[0.018]	[0.209]	[0.436]	[0.172]	[0.003]	[0.745]	[0.101]	[0.314]
Mixed Crops (dummy)	3.469	185.109***	-1.173***	-13.518	70.688	-0.086	6.356	124.526**	0.032***
	(35.432)	(68.250)	(0.177)	(18.699)	(56.364)	(0.147)	(23.262)	(52.898)	(0.007)
	[0.922]	[0.007]	[0.000]	[0.470]	[0.210]	[0.559]	[0.785]	[0.019]	[0.000]
Labor force (man.day)	0.420	-17.061**	0.083***	-0.815	-10.076*	0.048***	-1.814	-1.984	0.002***
	(3.724)	(7.625)	(0.020)	(1.708)	(5.201)	(0.014)	(1.575)	(3.584)	(0.000)
	[0.910]	[0.025]	[0.000]	[0.634]	[0.053]	[0.000]	[0.249]	[0.580]	[0.000]
Unit selling price (USDkg)	-237.000	-721.133*	0.285	123.920	-92.826	-1.312	120.147	-218.466	0.021
	(180.874)	(413.285)	(1.069)	(142.336)	(521.062)	(1.358)	(95.949)	(246.713)	(0.034)
	[0.190]	[0.081]	[0.790]	[0.384]	[0.859]	[0.334]	[0.210]	[0.376]	[0.545]
Seed cost (USDha)	-0.637	-3.085	0.025***	0.213	0.171	0.001	-0.379	-6.068***	0.001***
	(0.987)	(2.125)	(0.005)	(0.409)	(1.243)	(0.003)	(0.755)	(1.715)	(0.000)
	[0.519]	[0.146]	[0.000]	[0.603]	[0.891]	[0.712]	[0.616]	[0.000]	[0.001]
Fertilizer cost (USDha)	-4.598	-4.762	0.000	-0.272	-7.616***	0.016***	-0.055	-3.677***	0.000
	(3.842)	(9.522)	(0.025)	(0.678)	(2.340)	(0.006)	(0.323)	(0.738)	(0.000)
	[0.231]	[0.617]	[0.990]	[0.689]	[0.001]	[0.008]	[0.866]	[0.000]	[0.344]
Pesticide cost (USDha)	-1.032	0.639	-0.034**	1.097	2.932	0.007	0.130	-1.656	-0.000
	(2.706)	(5.248)	(0.014)	(1.133)	(3.549)	(0.009)	(0.928)	(2.134)	(0.000)
	[0.703]	[0.903]	[0.012]	[0.333]	[0.409]	[0.447]	[0.889]	[0.438]	[0.622]
Labor cost (USDha)	-0.465	-1.213	0.002	-0.249	0.686	0.002	-0.005	-0.942	0.000
	(0.396)	(0.778)	(0.002)	(0.275)	(0.834)	(0.002)	(0.282)	(0.648)	(0.000)
	[0.240]	[0.119]	[0.326]	[0.367]	[0.411]	[0.434]	[0.985]	[0.146]	[0.104]
Groundnut area (ha)	3.858	374.767***	-0.092	10.579*	338.827***	0.283***	-3.431	380.566***	-0.004
	(14.086)	(27.147)	(0.070)	(5.695)	(17.181)	(0.045)	(9.833)	(22.397)	(0.003)
	[0.784]	[0.000]	[0.192]	[0.063]	[0.000]	[0.000]	[0.727]	[0.000]	[0.242]
Off-farm income (dummy)	59.648	9.799	-0.977**	5.396	-95.190	-0.559	-51.022*	-126.930**	-0.001
	(94.453)	(181.819)	(0.470)	(67.896)	(204.691)	(0.534)	(27.937)	(63.674)	(0.009)
	[0.528]	[0.957]	[0.038]	[0.937]	[0.642]	[0.294]	[0.068]	[0.046]	[0.951]
Dependency ratio	-2.013	39.761	-0.071	-4.460	13.217	-0.139**	-2.709	-16.735	0.008***
	(14.110)	(27.090)	(0.070)	(8.003)	(24.154)	(0.063)	(7.012)	(15.949)	(0.002)
	[0.887]	[0.142]	[0.308]	[0.577]	[0.584]	[0.027]	[0.699]	[0.294]	[0.000]
Clay soil (dummy)	29.747	35.638	0.007	5.276	2.513	-0.397*	-3.748	-184.403**	-0.008
	(32.359)	(84.400)	(0.218)	(18.778)	(79.547)	(0.207)	(21.723)	(73.064)	(0.010)
	[0.358]	[0.673]	[0.974]	[0.779]	[0.975]	[0.056]	[0.863]	[0.012]	[0.413]
Sandy-clay soil (dummy)	7.325	13.669	0.064	-0.860	-88.687	0.115	-6.272	-93.552*	-0.000
	(26.595)	(70.846)	(0.183)	(15.925)	(66.068)	(0.172)	(16.916)	(54.409)	(0.007)
	[0.783]	[0.847]	[0.727]	[0.957]	[0.179]	[0.502]	[0.711]	[0.086]	[0.971]
Silty soil (dummy)	59.965*	126.467	0.005	15.449	63.653	-0.091	-1.606	-90.246	-0.010
	(33.349)	(94.579)	(0.245)	(18.523)	(78.008)	(0.203)	(21.510)	(70.628)	(0.010)
	[0.072]	[0.181]	[0.985]	[0.404]	[0.415]	[0.653]	[0.940]	[0.201]	[0.305]
Constant	976.699***	-213.074	2.079	393.419***	959.988	-7.352	544.008***	-1,882.723*	0.992***
	(160.862)	(1,758.803)	(4.549)	(126.579)	(2,434.762)	(6.346)	(128.372)	(1,070.511)	(0.147)
	[0.000]	[0.904]	[0.648]	[0.002]	[0.693]	[0.247]	[0.000]	[0.079]	[0.000]
Observations	1,494	1,494	1,494	2,520	2,520	2,520	4,590	4,590	4,590
Number of id	498	498	498	840	840	840	1,530	1,530	1,530
District FE	YES	YES	YES	YES	YES	YES	YES	YES	YES

Table S10: 2SLS estimates of the relationship between adoption and commercialization and yield across countries *(continued)*

variables	Yield	Consumption	Sales value	Yield	Consumption	Sales value	Yield	Consumption	Sales value
Year FE	YES	YES	YES	YES	YES	YES	YES	YES	YES
Standard errors in brackets									
*** p<0.01, ** p<0.05, * p<0.1									

Note: The table presents the results of 2SLS regressions, which examine the relationship between adoption decision and various factors associated with Yield (1), Consumption (2), and Sales value (3) across the three study countries. The regressions employed Random Effect (RE) specifications for the yield variable, while Fixed Effect (FE) specifications were used for the Sales value and consumption variables. Robust standard errors are reported in brackets. Two-sided t-tests were employed for the statistical tests, and p-values are indicated within square brackets. Coefficients marked with an asterisk (*) signify statistical significance at predetermined levels of significance (*** p<0.01, ** p<0.05, * p<0.1). To account for potential unobserved heterogeneity, all regressions include a comprehensive set of district fixed effects.

Table S11: 2SLS estimates of the relationship between adoption and commercialization, production and yield simultaneously

	(1)	(2)	(3)
variables	Market participation	Quantity sold	Sales value
Total quantity of groundnut harvested	0.000***	0.000***	0.000***
	(0.000)	(0.000)	(0.000)
	[0.000]	[0.000]	[0.000]
Household groundnut consoumption	-0.000***	-0.001***	-0.001***
	(0.000)	(0.000)	(0.000)
	[0.000]	[0.000]	[0.000]
Age of household head (years)	-0.001	0.012	0.003
	(0.000)	(0.023)	(0.021)
	[0.119]	[0.604]	[0.867]
Sex of household head (dummy, male=1)	-0.013		
	(0.020)		
	[0.521]		
Education level (Number of years)	0.001		
	(0.001)		
	[0.312]		
Household size (number of persons)	0.000	0.020***	0.020***
	(0.001)	(0.005)	(0.004)
	[0.485]	[0.000]	[0.000]
Farmers group membership (dummy)	0.024***	0.146***	0.128***
	(0.004)	(0.033)	(0.030)
	[0.000]	[0.000]	[0.000]
Training on agriculture (dummy)	-0.051***	-0.275***	-0.259***
	(0.009)	(0.074)	(0.068)
	[0.000]	[0.000]	[0.000]
Training on groundnut farming (dummy)	-0.021***	-0.154***	-0.136***
	(0.003)	(0.022)	(0.020)
	[0.000]	[0.000]	[0.000]
Public agricultural extension service (number of visits)	0.004*	0.003	-0.004
	(0.002)	(0.016)	(0.014)
	[0.052]	[0.849]	[0.771]
Private agricultural extension service (number of visits)	0.004	0.051**	0.032
	(0.003)	(0.022)	(0.021)
	[0.206]	[0.022]	[0.122]
Cash credit for groundnut farming (dummy)	0.004	-0.075	-0.118
	(0.020)	(0.149)	(0.137)
	[0.822]	[0.614]	[0.389]
Credit in kind for groundnut farming (dummy)	-0.028**	-0.038	-0.039
	(0.013)	(0.104)	(0.096)
	[0.036]	[0.717]	[0.681]
Distance to the nearest urban market (km)	-0.001***	-0.002	-0.002
	(0.000)	(0.002)	(0.002)
	[0.000]	[0.257]	[0.336]
Distance the nearest village market (km)	-0.003***	-0.012*	-0.011**
	(0.001)	(0.006)	(0.006)
	[0.000]	[0.053]	[0.045]
Crop rotation (dummy)	-0.001	-0.094	-0.059
	(0.009)	(0.069)	(0.063)
	[0.936]	[0.173]	[0.356]
Mixed Crops (dummy)	0.010	0.010	0.006
	(0.007)	(0.058)	(0.053)
	[0.156]	[0.858]	[0.909]
Labor force (man.day)	0.002***	0.023***	0.023***
	(0.001)	(0.004)	(0.004)
	[0.003]	[0.000]	[0.000]
Unit selling price (USDkg)	0.012	0.796***	1.244***
	(0.049)	(0.222)	(0.322)
	[0.805]	[0.000]	[0.000]
Seed cost (USDha)	0.001***	0.010***	0.008***
	(0.000)	(0.001)	(0.001)
	[0.000]	[0.000]	[0.000]
Fertilizer cost (USDha)	-0.000	-0.001	-0.001
	(0.000)	(0.001)	(0.001)
	[0.405]	[0.438]	[0.218]

Table S11: 2SLS estimates of the relationship between adoption and commercialization, production and yield simultaneously *(continued)*

variables	Market participation	Quantity sold	Sales value
Pesticide cost (USDha)	-0.001***	-0.003	-0.005*
	(0.000)	(0.003)	(0.003)
	[0.004]	[0.217]	[0.061]
Labor cost (USDha)	0.000***	0.002**	0.002**
	(0.000)	(0.001)	(0.001)
	[0.000]	[0.031]	[0.026]
Groundnut area (ha)	0.013***	0.176***	0.187***
	(0.004)	(0.028)	(0.026)
	[0.000]	[0.000]	[0.000]
Off-farm income (dummy)	-0.032***	-0.074	-0.065
	(0.012)	(0.094)	(0.086)
	[0.008]	[0.431]	[0.448]
Dependency ratio	0.001	0.014	0.014
	(0.003)	(0.021)	(0.019)
	[0.689]	[0.513]	[0.454]
Clay soil (dummy)	-0.015	-0.222***	-0.206***
	(0.011)	(0.083)	(0.076)
	[0.144]	[0.008]	[0.007]
Sandy-clay soil (dummy)	0.002	0.000	-0.001
	(0.008)	(0.065)	(0.060)
	[0.830]	[0.998]	[0.993]
Silty soil (dummy)	-0.001	-0.052	-0.056
	(0.011)	(0.083)	(0.076)
	[0.952]	[0.527]	[0.458]
Constant	0.950***	4.123***	4.856***
	(0.052)	(1.177)	(1.082)
	[0.000]	[0.000]	[0.000]
Observations	8,604	8,604	8,604
R-squared		0.172	
Number of id	2,868	2,868	2,868
District FE	YES	YES	YES
Year FE	YES	YES	YES
Standard errors in brackets			
*** p<0.01, ** p<0.05, * p<0.1			

Note: The table presents the results of 2SLS regressions, which examine the relationship between production and consumption of Groundnuts and various factors associated with Market participation (1), quantity sold (2), and Sales value (3). Robust standard errors are reported in brackets. Two-sided t-tests were employed for the statistical tests, and p-values are indicated within square brackets. Coefficients marked with an asterisk (*) signify statistical significance at predetermined levels of significance (*** p<0.01, ** p<0.05, * p<0.1). To account for potential unobserved heterogeneity, all regressions include a comprehensive set of district fixed effects.

2 Robustness checks

2.1 Control function approach

Table S12: Control function estimations of the relationship between adoption and market participation

	Market participation		Quantity sold		Sales value	
variables	FE	CRE	FE	CRE	FE	CRE
Adoption dummy	0.063***	0.050***	0.588***	0.520***	0.565***	0.503***
	(0.020)	(0.018)	(0.133)	(0.119)	(0.122)	(0.109)
	[0.001]	[0.004]	[0.000]	[0.000]	[0.000]	[0.000]
Age of household head (years)	0.002	0.001	-0.010	-0.016	-0.013	-0.018
	(0.003)	(0.003)	(0.024)	(0.024)	(0.022)	(0.022)
	[0.539]	[0.701]	[0.660]	[0.490]	[0.565]	[0.409]
Sex of household head (dummy, male=1)		-0.014		0.071		0.079
		(0.020)		(0.139)		(0.127)
		[0.481]		[0.611]		[0.533]
Education level (Number of years)		0.001		-0.005		-0.005

Table S12: Control function estimations of the relationship between adoption and market participation *(continued)*

variables	FE	CRE	FE	CRE	FE	CRE
		(0.001)		(0.009)		(0.008)
		[0.601]		[0.603]		[0.533]
Household size (number of persons)	0.002***	0.002***	0.027***	0.027***	0.026***	0.026***
	(0.001)	(0.001)	(0.005)	(0.005)	(0.004)	(0.004)
	[0.004]	[0.005]	[0.000]	[0.000]	[0.000]	[0.000]
Farmers group membership (dummy)	0.022***	0.023***	0.124***	0.125***	0.111***	0.111***
	(0.005)	(0.005)	(0.035)	(0.035)	(0.032)	(0.032)
	[0.000]	[0.000]	[0.000]	[0.000]	[0.000]	[0.000]
Training on agriculture (dummy)	-0.043***	-0.041***	-0.314***	-0.298***	-0.287***	-0.273***
	(0.011)	(0.011)	(0.078)	(0.078)	(0.071)	(0.072)
	[0.000]	[0.000]	[0.000]	[0.000]	[0.000]	[0.000]
Training on groundnut farming (dummy)	-0.025***	-0.025***	-0.176***	-0.178***	-0.162***	-0.165***
	(0.003)	(0.003)	(0.023)	(0.023)	(0.021)	(0.021)
	[0.000]	[0.000]	[0.000]	[0.000]	[0.000]	[0.000]
Public agricultural extension service (number of visits)	0.002	0.003	-0.024	-0.018	-0.025	-0.020
	(0.002)	(0.002)	(0.016)	(0.016)	(0.015)	(0.015)
	[0.355]	[0.190]	[0.148]	[0.265]	[0.102]	[0.191]
Private agricultural extension service (number of visits)	0.004	0.003	0.045*	0.042*	0.042*	0.040*
	(0.003)	(0.003)	(0.024)	(0.023)	(0.022)	(0.022)
	[0.313]	[0.334]	[0.057]	[0.071]	[0.052]	[0.066]
Cash credit for groundnut farming (dummy)	-0.010	-0.008	-0.188	-0.175	-0.194	-0.182
	(0.023)	(0.023)	(0.156)	(0.156)	(0.143)	(0.143)
	[0.659]	[0.729]	[0.229]	[0.261]	[0.176]	[0.202]
Credit in kind for groundnut farming (dummy)	-0.044***	-0.043***	-0.046	-0.040	-0.022	-0.017
	(0.016)	(0.016)	(0.109)	(0.109)	(0.100)	(0.100)
	[0.007]	[0.008]	[0.675]	[0.712]	[0.829]	[0.867]
Distance to the nearest urban market (km)	-0.000	-0.001***	-0.003*	-0.009***	-0.003*	-0.008***
	(0.000)	(0.000)	(0.002)	(0.002)	(0.002)	(0.002)
	[0.146]	[0.000]	[0.089]	[0.000]	[0.087]	[0.000]
Distance the nearest village market (km)	-0.003***	-0.003***	-0.013**	-0.017***	-0.011*	-0.015***
	(0.001)	(0.001)	(0.006)	(0.005)	(0.006)	(0.005)
	[0.003]	[0.000]	[0.044]	[0.002]	[0.053]	[0.002]
Crop rotation (dummy)	-0.023**	-0.021**	-0.146**	-0.128*	-0.136**	-0.119*
	(0.011)	(0.011)	(0.072)	(0.072)	(0.066)	(0.066)
	[0.029]	[0.043]	[0.044]	[0.076]	[0.041]	[0.072]
Mixed Crops (dummy)	0.004	0.001	-0.062	-0.079	-0.065	-0.080
	(0.009)	(0.009)	(0.061)	(0.061)	(0.056)	(0.056)
	[0.673]	[0.932]	[0.304]	[0.190]	[0.243]	[0.149]
Labor force (man.day)	0.002***	0.002***	0.027***	0.027***	0.026***	0.026***
	(0.001)	(0.001)	(0.005)	(0.005)	(0.004)	(0.004)
	[0.003]	[0.003]	[0.000]	[0.000]	[0.000]	[0.000]
Unit selling price (USDkg)	0.010	0.033	-0.122	-0.001	1.313***	1.426***
	(0.053)	(0.050)	(0.362)	(0.341)	(0.333)	(0.313)
	[0.859]	[0.514]	[0.737]	[0.998]	[0.000]	[0.000]
Seed cost (USDha)	0.002***	0.002***	0.011***	0.010***	0.010***	0.009***
	(0.000)	(0.000)	(0.002)	(0.002)	(0.001)	(0.001)
	[0.000]	[0.000]	[0.000]	[0.000]	[0.000]	[0.000]
Fertilizer cost (USDha)	0.000	0.000	0.001	0.001	0.001	0.001
	(0.000)	(0.000)	(0.001)	(0.001)	(0.001)	(0.001)
	[0.724]	[0.573]	[0.206]	[0.174]	[0.219]	[0.189]
Pesticide cost (USDha)	-0.001***	-0.001***	-0.005	-0.006*	-0.004	-0.005*
	(0.000)	(0.000)	(0.003)	(0.003)	(0.003)	(0.003)
	[0.004]	[0.002]	[0.108]	[0.054]	[0.138]	[0.071]
Labor cost (USDha)	0.000***	0.000***	0.002***	0.002***	0.002***	0.002***
	(0.000)	(0.000)	(0.001)	(0.001)	(0.001)	(0.001)
	[0.001]	[0.000]	[0.004]	[0.002]	[0.004]	[0.003]
Groundnut area (ha)	0.003	0.004	0.198***	0.204***	0.195***	0.201***
	(0.003)	(0.003)	(0.023)	(0.023)	(0.021)	(0.021)
	[0.410]	[0.282]	[0.000]	[0.000]	[0.000]	[0.000]
Off-farm income (dummy)	-0.020	-0.021	-0.035	-0.044	-0.024	-0.032
	(0.014)	(0.014)	(0.098)	(0.098)	(0.090)	(0.090)
	[0.173]	[0.139]	[0.717]	[0.651]	[0.787]	[0.720]
Dependency ratio	0.002	0.002	0.013	0.016	0.012	0.015
	(0.003)	(0.003)	(0.022)	(0.022)	(0.020)	(0.020)
	[0.609]	[0.529]	[0.557]	[0.462]	[0.551]	[0.456]

Table S12: Control function estimations of the relationship between adoption and market participation *(continued)*

variables	FE	CRE	FE	CRE	FE	CRE
Clay soil (dummy)	-0.016	-0.013	-0.160*	-0.129*	-0.154*	-0.125*
	(0.013)	(0.011)	(0.087)	(0.073)	(0.080)	(0.067)
	[0.203]	[0.227]	[0.066]	[0.077]	[0.055]	[0.063]
Sandy-clay soil (dummy)	0.006	0.006	0.052	0.039	0.048	0.036
	(0.010)	(0.009)	(0.068)	(0.059)	(0.063)	(0.054)
	[0.526]	[0.486]	[0.448]	[0.503]	[0.449]	[0.507]
Silty soil (dummy)	-0.004	0.001	-0.032	0.010	-0.032	0.007
	(0.013)	(0.011)	(0.086)	(0.073)	(0.079)	(0.067)
	[0.735]	[0.899]	[0.715]	[0.892]	[0.684]	[0.915]
Constant	0.700***	1.021***	5.500***	7.367***	4.331***	6.012***
	(0.183)	(0.076)	(1.242)	(0.521)	(1.142)	(0.478)
	[0.000]	[0.000]	[0.000]	[0.000]	[0.000]	[0.000]
Observations	8,604	8,604	8,604	8,604	8,604	8,604
R-squared	0.066		0.098		0.126	
Number of id	2,868	2,868	2,868	2,868	2,868	2,868
District FE	YES	YES	YES	YES	YES	YES
Year FE	YES	YES	YES	YES	YES	YES
Standard errors in brackets						
*** p<0.01, ** p<0.05, * p<0.1						

Note: The table provides the results of ontrol function estimations, which investigates the relationship between adoption and various factors related to Market participation, quantity sold, and Sales value under both Fixed effects (FE) and corrolated randome effect (CRE) specifications. Robust standard errors are reported in brackets to account for potential heteroscedasticity. Two-sided t-tests were used for statistical testing, and the corresponding p-values are presented within square brackets. Coefficients marked with an asterisk (*) indicate statistical significance at predetermined levels of significance (*** p<0.01, ** p<0.05, * p<0.1). To control for potential unobserved heterogeneity, all regressions incorporate a comprehensive set of district fixed effects.

2.2 Hausman Taylor IV

Table S13: HAUSMAN TAYLOR IV estimations

variables	Market participation	Quantity sold	Sales value
Adoption dummy	0.038***	0.279***	0.258***
	(0.012)	(0.081)	(0.074)
	[0.001]	[0.001]	[0.001]
Age of household head (years)	-0.002***	-0.016***	-0.015***
	(0.000)	(0.004)	(0.003)
	[0.001]	[0.000]	[0.000]
Household size (number of persons)	0.000	0.012***	0.012***
	(0.001)	(0.004)	(0.004)
	[0.409]	[0.002]	[0.001]
Farmers group membership (dummy)	0.007	0.019	0.015
	(0.004)	(0.029)	(0.027)
	[0.112]	[0.510]	[0.562]
Training on agriculture (dummy)	-0.049***	-0.311***	-0.281***
	(0.009)	(0.066)	(0.061)
	[0.000]	[0.000]	[0.000]
Training on groundnut farming (dummy)	-0.009***	-0.038*	-0.034*
	(0.003)	(0.020)	(0.018)
	[0.003]	[0.061]	[0.067]
Public agricultural extension service (number of visits)	-0.005**	-0.062***	-0.059***
	(0.002)	(0.014)	(0.013)
	[0.011]	[0.000]	[0.000]
Private agricultural extension service (number of visits)	-0.000	0.004	0.002
	(0.003)	(0.020)	(0.018)
	[0.975]	[0.861]	[0.905]
Cash credit for groundnut farming (dummy)	0.014	-0.027	-0.043
	(0.020)	(0.140)	(0.128)
	[0.492]	[0.848]	[0.737]
Credit in kind for groundnut farming (dummy)	-0.037***	0.003	0.023
	(0.014)	(0.096)	(0.088)
	[0.008]	[0.974]	[0.797]
Distance to the nearest urban market (km)	-0.000	-0.000	-0.000
	(0.000)	(0.002)	(0.002)
	[0.135]	[0.807]	[0.895]
Distance the nearest village market (km)	-0.004***	-0.021***	-0.019***
	(0.001)	(0.005)	(0.005)
	[0.000]	[0.000]	[0.000]
Crop rotation (dummy)	-0.050***	-0.487***	-0.461***
	(0.008)	(0.058)	(0.054)
	[0.000]	[0.000]	[0.000]
Mixed Crops (dummy)	0.018**	0.049	0.039
	(0.007)	(0.051)	(0.047)
	[0.016]	[0.343]	[0.410]
Labor force (man.day)	0.003***	0.035***	0.033***
	(0.001)	(0.004)	(0.004)
	[0.000]	[0.000]	[0.000]
Unit selling price (USDkg)	0.093**	0.867***	2.280***
	(0.041)	(0.277)	(0.255)
	[0.024]	[0.002]	[0.000]
Seed cost (USDha)	0.002***	0.012***	0.011***
	(0.000)	(0.001)	(0.001)
	[0.000]	[0.000]	[0.000]
Fertilizer cost (USDha)	0.000***	0.005***	0.005***
	(0.000)	(0.001)	(0.001)
	[0.000]	[0.000]	[0.000]
Pesticide cost (USDha)	-0.001***	-0.000	0.001
	(0.000)	(0.003)	(0.002)
	[0.010]	[0.997]	[0.787]
Labor cost (USDha)	0.000***	0.001	0.001
	(0.000)	(0.001)	(0.001)
	[0.008]	[0.169]	[0.197]
Groundnut area (ha)	0.019***	0.326***	0.314***
	(0.003)	(0.019)	(0.018)
	[0.000]	[0.000]	[0.000]

Table S13: HAUSMAN TAYLOR IV estimations *(continued)*

variables	Market participation	Quantity sold	Sales value
Off-farm income (dummy)	-0.017	0.021	0.030
	(0.012)	(0.084)	(0.077)
	[0.159]	[0.806]	[0.698]
Dependency ratio	0.006**	0.033*	0.030*
	(0.003)	(0.019)	(0.017)
	[0.038]	[0.080]	[0.084]
Clay soil (dummy)	-0.020*	-0.182**	-0.174**
	(0.011)	(0.077)	(0.071)
	[0.079]	[0.018]	[0.014]
Sandy-clay soil (dummy)	0.004	0.034	0.031
	(0.009)	(0.061)	(0.056)
	[0.675]	[0.579]	[0.576]
Silty soil (dummy)	-0.002	-0.025	-0.026
	(0.011)	(0.077)	(0.071)
	[0.848]	[0.747]	[0.716]
Sex of household head (dummy, male=1)	0.044**	0.745***	0.719***
	(0.022)	(0.167)	(0.154)
	[0.047]	[0.000]	[0.000]
Education level (Number of years)	0.004***	0.026**	0.024**
	(0.001)	(0.011)	(0.010)
	[0.005]	[0.013]	[0.014]
Constant	0.783***	4.838***	3.564***
	(0.041)	(0.294)	(0.271)
	[0.000]	[0.000]	[0.000]
Observations	8,604	8,604	8,604
Number of id	2,868	2,868	2,868
Standard errors in brackets			
*** p<0.01, ** p<0.05, * p<0.1			

Note: The table provides the results of the HAUSMAN TAYLOR IV model, which investigates the relationship between adoption and various factors related to Market participation, quantity sold, and Sales value. Robust standard errors are reported in brackets to account for potential heteroscedasticity. Two-sided t-tests were used for statistical testing, and the corresponding p-values are presented within square brackets. Coefficients marked with an asterisk (*) indicate statistical significance at predetermined levels of significance (*** p<0.01, ** p<0.05, * p<0.1). To control for potential unobserved heterogeneity, all regressions incorporate a comprehensive set of district fixed effects.

2.3 Lewbels Test

Table S14: Lewbel instrumental variable estimations of the relationship between adoption and commercialization

				Acess (IV)		
variables	Market participation	Quantity sold	Sales value	Market participation	Quantity sold	Sales value
Adoption dummy	0.043***	0.315***	0.291***	0.043***	0.315***	0.291***
	(0.013)	(0.087)	(0.080)	(0.013)	(0.087)	(0.080)
	[0.001]	[0.000]	[0.000]	[0.001]	[0.000]	[0.000]
Age of household head	0.001	-0.018	-0.019	0.001	-0.018	-0.019
	(0.003)	(0.024)	(0.022)	(0.003)	(0.024)	(0.022)
	[0.864]	[0.450]	[0.383]	[0.864]	[0.450]	[0.383]
Household size	0.002**	0.027***	0.026***	0.002**	0.027***	0.026***
	(0.001)	(0.007)	(0.006)	(0.001)	(0.007)	(0.006)
	[0.036]	[0.000]	[0.000]	[0.036]	[0.000]	[0.000]
Farmers group membership	0.023***	0.132***	0.118***	0.023***	0.132***	0.118***
	(0.006)	(0.044)	(0.041)	(0.006)	(0.044)	(0.041)
	[0.000]	[0.003]	[0.004]	[0.000]	[0.003]	[0.004]
Training on agriculture	-0.042***	-0.300***	-0.274***	-0.042***	-0.300***	-0.274***
	(0.015)	(0.105)	(0.096)	(0.015)	(0.105)	(0.096)
	[0.006]	[0.004]	[0.004]	[0.006]	[0.004]	[0.004]
Training on groundnut farming	-0.025***	-0.179***	-0.166***	-0.025***	-0.179***	-0.166***
	(0.005)	(0.031)	(0.028)	(0.005)	(0.031)	(0.028)
	[0.000]	[0.000]	[0.000]	[0.000]	[0.000]	[0.000]
Public agricultural extension service	0.003	-0.018	-0.019	0.003	-0.018	-0.019
	(0.003)	(0.019)	(0.018)	(0.003)	(0.019)	(0.018)
	[0.293]	[0.352]	[0.282]	[0.293]	[0.352]	[0.282]
Private agricultural extension service	0.003	0.044	0.042	0.003	0.044	0.042
	(0.004)	(0.029)	(0.027)	(0.004)	(0.029)	(0.027)
	[0.476]	[0.128]	[0.116]	[0.476]	[0.128]	[0.116]
Cash credit for groundnut farming	-0.006	-0.150	-0.158	-0.006	-0.150	-0.158
	(0.027)	(0.189)	(0.174)	(0.027)	(0.189)	(0.174)
	[0.835]	[0.427]	[0.366]	[0.835]	[0.427]	[0.366]
Credit in kind for groundnut farming	-0.043**	-0.036	-0.012	-0.043**	-0.036	-0.012
	(0.019)	(0.133)	(0.124)	(0.019)	(0.133)	(0.124)
	[0.025]	[0.784]	[0.925]	[0.025]	[0.784]	[0.925]
Distance to the nearest urban market	-0.000	-0.003*	-0.003*	-0.000	-0.003*	-0.003*
	(0.000)	(0.002)	(0.002)	(0.000)	(0.002)	(0.002)
	[0.146]	[0.087]	[0.084]	[0.146]	[0.087]	[0.084]
Distance the nearest village market	-0.002	-0.009	-0.008	-0.002	-0.009	-0.008
	(0.002)	(0.011)	(0.010)	(0.002)	(0.011)	(0.010)
	[0.131]	[0.393]	[0.421]	[0.131]	[0.393]	[0.421]
Crop rotation	-0.022	-0.136	-0.128	-0.022	-0.136	-0.128
	(0.014)	(0.088)	(0.081)	(0.014)	(0.088)	(0.081)
	[0.112]	[0.122]	[0.111]	[0.112]	[0.122]	[0.111]
Mixed Crops	0.001	-0.081	-0.082	0.001	-0.081	-0.082
	(0.011)	(0.078)	(0.072)	(0.011)	(0.078)	(0.072)
	[0.949]	[0.297]	[0.250]	[0.949]	[0.297]	[0.250]
Labor force	0.002***	0.026***	0.025***	0.002***	0.026***	0.025***
	(0.001)	(0.005)	(0.005)	(0.001)	(0.005)	(0.005)
	[0.004]	[0.000]	[0.000]	[0.004]	[0.000]	[0.000]
Unit selling price	0.050	0.452	1.890***	0.050	0.452	1.890***
	(0.043)	(0.291)	(0.268)	(0.043)	(0.291)	(0.268)
	[0.249]	[0.120]	[0.000]	[0.249]	[0.120]	[0.000]
Seed cost	0.002***	0.011***	0.010***	0.002***	0.011***	0.010***
	(0.000)	(0.002)	(0.002)	(0.000)	(0.002)	(0.002)
	[0.000]	[0.000]	[0.000]	[0.000]	[0.000]	[0.000]
Fertilizer cost	0.000	0.002	0.001	0.000	0.002	0.001
	(0.000)	(0.001)	(0.001)	(0.000)	(0.001)	(0.001)
	[0.505]	[0.106]	[0.117]	[0.505]	[0.106]	[0.117]
Pesticide cost	-0.001***	-0.004	-0.003	-0.001***	-0.004	-0.003
	(0.000)	(0.003)	(0.003)	(0.000)	(0.003)	(0.003)
	[0.007]	[0.218]	[0.280]	[0.007]	[0.218]	[0.280]
Labor cost	0.000***	0.002**	0.002**	0.000***	0.002**	0.002**
	(0.000)	(0.001)	(0.001)	(0.000)	(0.001)	(0.001)
	[0.004]	[0.011]	[0.011]	[0.004]	[0.011]	[0.011]
Groundnut area	0.004	0.208***	0.204***	0.004	0.208***	0.204***

Table S14: Lewbel instrumental variable estimations of the relationship between adoption and commercialization *(continued)*

variables	Market participation	Quantity sold	Sales value	Market participation	Quantity sold	Sales value
	(0.004)	(0.031)	(0.029)	(0.004)	(0.031)	(0.029)
	[0.298]	[0.000]	[0.000]	[0.298]	[0.000]	[0.000]
Off-farm income	-0.020	-0.036	-0.024	-0.020	-0.036	-0.024
	(0.014)	(0.106)	(0.099)	(0.014)	(0.106)	(0.099)
	[0.168]	[0.737]	[0.809]	[0.168]	[0.737]	[0.809]
Dependency ratio	0.001	0.011	0.010	0.001	0.011	0.010
	(0.004)	(0.025)	(0.023)	(0.004)	(0.025)	(0.023)
	[0.693]	[0.663]	[0.657]	[0.693]	[0.663]	[0.657]
Clay soil	-0.019	-0.173	-0.165*	-0.019	-0.173	-0.165*
	(0.016)	(0.109)	(0.100)	(0.016)	(0.109)	(0.100)
	[0.237]	[0.111]	[0.098]	[0.237]	[0.111]	[0.098]
Sandy-clay soil	0.004	0.037	0.034	0.004	0.037	0.034
	(0.012)	(0.082)	(0.075)	(0.012)	(0.082)	(0.075)
	[0.768]	[0.654]	[0.651]	[0.768]	[0.654]	[0.651]
Silty soil	-0.008	-0.047	-0.045	-0.008	-0.047	-0.045
	(0.015)	(0.104)	(0.096)	(0.015)	(0.104)	(0.096)
	[0.608]	[0.656]	[0.641]	[0.608]	[0.656]	[0.641]
Observations	8,604	8,604	8,604	8,604	8,604	8,604
R-squared	0.051	0.085	0.114	0.051	0.085	0.114
Robust standard errors in brackets						
*** p<0.01, ** p<0.05, * p<0.1						

Note: The table provides the results of Lewbel instrumental variable (IV) estimations, which investigate the relationship between adoption and several factors associated with market participation, quantity sold, and sales value. The Lewbel IV approach extends the standard instrumental variable technique by addressing the endogeneity issue that arises when the adoption decision and commercialization are jointly determined. Robust standard errors are reported in brackets to account for potential heteroscedasticity and model misspecification. Statistical tests were conducted using two-sided t-tests, and p-values are presented within square brackets. Coefficients marked with an asterisk (*) indicate statistical significance at predetermined levels of significance (*** p<0.01, ** p<0.05, * p<0.1). Additionally, to control for potential unobserved heterogeneity, all regressions incorporate a comprehensive set of district fixed effects, capturing the district-specific characteristics that may affect the adoption and market outcomes.

3 Questionnaire

Increasing Groundnut Productivity of Smallholder farmers in Ghana, Mali and Nigeria

---

Questionnaire for gross margin data collection

SECTION A. IDENTIFICATION OF THE ADMINISTRATIVE UNIT OF RESPONDENT

1- Country: [____] Nigeria = 3

2- State (Jigawa = 1, Kebbi = 2, Kano = 3, Katsina = 4, Sokoto = 5): ___________________________

3- Local Government Area (LGA):_____________________________________

4- Village: ___________________________

SECTION B. SOCIO-DEMOGRAPHIC CHARACTERISTICS OF THE HOUSEHOLD MEMBERS

5- Household respondent name

5.1- First name of household respondent ____

5.2- Family name of household respondent ____

5.3- Phone number of household respondent ____

6- Household characteristics

Note: *Household is defined as a group of people (related or not) who eat and live together in the same home for at least six months in a year. This includes the employees and visitors provided that they eat and live with one family most of the time. However, the household excludes the family members who live elsewhere most of the time, whether more than 6 months in a year or 4 days in a week.*

6.1. Sex of household head: Male = 1 Female = 0	
6.2. Age of household head (years)	
6.3. Marital status of household head: 1=single, 2=married, 3=widowed, 4=divorced, 5=other (specify)	
6.4. Number of children of age between 0 and 5 years	
6.5. Number of children of age between 6 and 14 years	
6.6. Number of persons of age between 15 and 35 years	
6.7. Number of persons of age between 36 and 54 years	
6.8. Number of persons of age between 55 and 64 years	
6.9. Number of persons of age greater than 65 years	
6.4. Number of years in agricultural activities as independent household head	
6.5. Number of years in groundnut production as independent household head	
6.6. Type of household agricultural farm: 1=commercial farm, 2=family farm (subsistence), 3=Predominantly subsistence with sale of surplus produce	

6.7. Enterprise mix of household : 1=groundnut + other crops, 0=only groundnut	
6.8. Do you or other members of your household belong to a cooperative or farming groups: 0=Not a member, 1=Yes, a farming group, 2=Yes, agriculture cooperative, 3=Yes, farming group and cooperative, 4=Other (specify)	
6.9. Have you or other members of your household received a training on agricultural production/marketing during the last cropping season : 0=no, 1=yes	
6.10. Have your or other members of your household received a training on groundnut production and aflatoxin management (delivered by this project or others, specify): 0=no, 1=yes	
6.11. Number of ADP extension visits related to agricultural production/marketing during the last cropping season	
6.12. Number of other extension visits related to agricultural production (NGO, cooperatives, etc.)	
6.13. Have you or any members of your household received credit during the last cropping season? 0=no, 1=yes	

7- Farm size

Production year	Household Total farm size (ha)	Total household farm size cultivated (ha)	Total farm size cultivated only by male (ha)	Total farm size cultivated only by female (ha)	Total farm size cultivated jointly by male and female (ha)	Total farm size cultivated by group (ha)

SECTION C. GROUNDNUT PRODUCTION COST

(i)

**Hectares planted**		**Total groundnut production**	
Total groundnut plots size (ha) managed by male?		Total groundnut production (kg) from plots managed by male?	
Total groundnut plots size (ha) managed by female?		Total groundnut production (kg) from plots managed by female?	
Total groundnut plots size (ha) managed jointly by male and female?		Total groundnut production (kg) from plots managed jointly by male and female?	
Total groundnut plots size (ha) managed in group?		Total groundnut production (kg) from plots managed in group?	

**Total quantity sold**		**Sales value**	
Total quantity of sales of the production managed by male (kg)?		Total value of sales of the production managed by male (Local Currency)?	
Total quantity of sales of the production managed by female (kg)?		Total value of sales of the production managed by female (Local Currency)?	
Total quantity of sales of the production managed jointly by male and female (kg)?		Total value of sales of the production managed jointly by male and female (Local Currency)?	
Total quantity of sales of the production managed in group (kg)?		Total value of sales of the production managed in group (Local Currency)?	

Seed cost		Fertilizer cost	
Total cost of seed (Local Currency) only by male		Total cost of fertilizer (Local Currency) only by male	
Total cost of seed (Local Currency) only by female		Total cost of fertilizer (Local Currency) only by female	
Total cost of seed (Local Currency) jointly by male and female		Total cost of fertilizer (Local Currency) jointly by male and female	
Total cost of seed (Local Currency) only by group, if production is done in farming groups		Total cost of fertilizer (Local Currency) only by group, if production is done in farming groups	

Pesticide cost		Hired labor cost	
Total cost of pesticides (Local Currency) only by male		Total cost of hired labor (Local Currency) only by male	
Total cost of pesticides (Local Currency) only by female		Total cost of hired labor (Local Currency) only by female	
Total cost of pesticides (Local Currency) jointly by male and female		Total cost of hired labor (Local Currency) jointly by male and female	
Total cost of pesticides (Local Currency a) only by group, if production is done in farming groups		Total cost of hired labor (Local Currency) only by group, if production is done in farming groups	

Hired services of farm equipment (including animal traction)				
Hired services of farm equipment (including animal traction)	Total cost of hired farm equipment including animal traction by male (Local currency)	Total cost of hired farm equipment including animal traction by female (Local currency)	Total cost of hired farm equipment including animal traction jointly by male and female (Local currency)	Total cost of hired farm equipment including animal traction in group farms (Local currency)

Household equipment used for groundnut production in 2017 (including animal traction)

	Number	Total cost of purchase	Year of purchase	lifetime
Hoe				
Chopped				
Wheelbarrows				
Cutlass				
Sprayer				
Tractor				
Motorcycle pump				
Seeders				
Pickaxe				
Harrow				
Plow				
Sheller				
Tumbrel				

Oxen				
Donkey				

SECTION D. GROUNDNUT VARIETIES USED DURING 2016 AND 2017

All groundnut varieties disseminated in the survey area (Code A)	Are you aware of this variety?	When did you know about it for the first time? (year)	Main source of information (Code B)	If you wish to grow this variety, can you have access to the seeds?		Did you cultivate this variety at least on time? (Code C)	Did you cultivate this variety in 2017? (Code C)	If no, why? (Code D)	If yes, what is the source of the first seeds used? (Code E)			Why do you continue to cultivate this variety? (Code F)
				In your village	Outside of your village				Source	Quantity	Unit	
Code A<br><br>1. SAMNUT 10 (RMP 12)<br>2. SAMNUT 11(RMP 91)<br>3. SAMNUT 21<br>4. SAMNUT 22<br>5. SAMNUT 23<br>6. SAMNUT 24<br>7. SAMNUT 25<br>8. SAMNUT 26<br>9. Maiborgo<br>10. Yardakar<br>11. Kampala<br>12. Manipinta<br>13. Kwarkwaso<br>14. Burguwa		**Code B**<br>1= Field trial/Field test/On-farm demonstration<br>2 = Open-door/meetings<br>3 = Another farmer/neighbor/family<br>4 = Extension services/NGO/research<br>5 = Cooperatives or Village Associations<br>6 = Rural radio/Postings/Brochures<br>7 = Other sources (specify)				**Code C**<br>1=Yes<br>2=No	**Code C**<br>1=Yes<br>2=No	**Code D**<br>1 = Seeds not available<br>2 = High cost of seeds<br>3 = Low yield<br>4 = Poor resistance to diseases<br>5 = Not suitable in association<br>6 = Poor drought resistance<br>7 = Poor resistance to insects<br>8 = Lack of information on the management of the variety<br>9 = Late Maturing<br>10 = Low market value of the variety<br>11 = Heavy fertilizer consumption<br>12 = not adapted to dietary habits<br>13 = Other reason (specify)	**Code E**<br>1= Field trial/Field test/On-farm demonstration<br>2= Another farmer/neighbor/family<br>3 = Clean seeds<br>4 = Seed salesman<br>5 = IAR/ICRISAT/ADP/IFAD<br>6 = Extension services/NGO<br>7 = Village cooperatives<br>8 = Seed companies<br>9 = Other source (specify)			**Code F**<br>1=Access to credit<br>2=Affiliation to a project<br>3=Access to equipment<br>4=Trained in groundnut production<br>5=Improved grain yield<br>6=Yield stability<br>7= Improved fodder yield<br>8= High oil content<br>9= For fresh nut sale<br>10=Others (specify)

SECTION E: DETAILS ON THE GROUNDNUT PLOTS

	Plot 1	Plot 2	Plot 3	Plot 4	Plot 5
Plot name/location (in order to find this plot next year if need)					
Farm size in 2017					
Name of the variety					
Type of land tenure (1 = land inherited, 2 = land received for rent, 3 = land received on free loan)					
Type of variety (1 = improved, 2 = local;)					
Sowing date					
Harvest date					
Soil type (1 = clay, 2 = sandy-clay, 3 = silty;)					
Evaluation of soil fertility (1 = good, 2 = average, 3 = bad;)					
Groundnut production in kg					
Evaluation of production (1 = good, 2 = average, 3 = bad;)					
Do you associate groundnut with other crop? (1 = yes, 0 = no)					
If yes, what kind of crop? (1 = maize, 2 = sorghum, 3 = millet, 4 = cowpea, 5 = cotton, 6 = other)					
Do you practice crop rotation?					
If yes, what was the previous crop? (1 = maize, 2 = sorghum, 3 = millet, 4 = cowpea, 5 = cotton, 6 = other)					
What is the quantity of seeds in kg used for the plot?					
What is the cost of the quantity of FCFA seed used for the plot?					
What is the quantity of fertilizer (NPK, Urea, PNT, DAP) in kg used for the plot?					
What is the cost of the amount of chemical fertilizer in local currency (LC) used for the plot?					
What is the amount of organic fertilizer in kg used for the plot?					
What is the cost of the amount of organic fertilizer in local currency (LC) used for the parcel?					
What is the quantity in liters of the herbicides used on the plot?					
What is the cost of herbicides used on the plot local currency (LC)?					
What is the quantity in liters of the fungicides used on the plot (L)?					
What is the cost of fungicides used on the plot local currency (LC)?					
What is the quantity in liter of pesticides used on the plot (L)?					
What is the cost of pesticides used on the plot local currency (LC)?					
How many cattle (oxen and donkeys) are used for agricultural work on the plot?					
What is the cost in local currency (LC) of using the animals on the plot?					
How many male did you use on the plot?					
For how many days men worked on this plot?					
What is the cost of the male labor force in local currency (LC) used on the plot?					
How many female did you use on the plot?					
For how many days women worked on this plot?					
What is the cost of female labor in local currency (LC) used on the plot?					

How many children did you use on the plot?					
For how many days children worked on this plot?					
What is the cost of children labor in local currency (LC) used on the plot?					
Person responsible for the plot (1 = head of household, 2 = wife, 3=son / daughter, 4 = Other parents, 5 = Other members)					
Sex of the responsible (1 = male, 0= female)					
Age of the responsible					
