# Supplementary material for: Adoption of climate-resilient groundnut varieties increases agricultural production, consumption, and smallholder commercialization in West Africa
Source: Nat Commun. 2023 Aug 24;14:5175. doi: 10.1038/s41467-023-40781-1 (PMC10449883; doi:10.1038/s41467-023-40781-1)
Supplement: Supplementary file 2 — Reporting Summary [file 41467_2023_40781_MOESM2_ESM.pdf]

nature portfolio

Corresponding author(s): Martin Paul Jr, Tabe-Ojong

Last updated by author(s): Jun 25, 2023

Reporting Summary

Nature Portfolio wishes to improve the reproducibility of the work that we publish. This form provides structure for consistency and transparency in reporting. For further information on Nature Portfolio policies, see our Editorial Policies and the Editorial Policy Checklist.

Statistics

For all statistical analyses, confirm that the following items are present in the figure legend, table legend, main text, or Methods section.

n/a	Confirmed	
☐	☒	The exact sample size (*n*) for each experimental group/condition, given as a discrete number and unit of measurement
☐	☒	A statement on whether measurements were taken from distinct samples or whether the same sample was measured repeatedly
☐	☒	The statistical test(s) used AND whether they are one- or two-sided<br>*Only common tests should be described solely by name; describe more complex techniques in the Methods section.*
☐	☒	A description of all covariates tested
☐	☒	A description of any assumptions or corrections, such as tests of normality and adjustment for multiple comparisons
☐	☒	A full description of the statistical parameters including central tendency (e.g. means) or other basic estimates (e.g. regression coefficient) AND variation (e.g. standard deviation) or associated estimates of uncertainty (e.g. confidence intervals)
☐	☒	For null hypothesis testing, the test statistic (e.g. *F*, *t*, *r*) with confidence intervals, effect sizes, degrees of freedom and *P* value noted<br>*Give P values as exact values whenever suitable.*
☒	☐	For Bayesian analysis, information on the choice of priors and Markov chain Monte Carlo settings
☒	☐	For hierarchical and complex designs, identification of the appropriate level for tests and full reporting of outcomes
☒	☐	Estimates of effect sizes (e.g. Cohen's *d*, Pearson's *r*), indicating how they were calculated

Our web collection on statistics for biologists contains articles on many of the points above.

Software and code

Policy information about availability of computer code

Data collection	Data were collected using a pre-tested questionnaire on CSPro 7.1.
Data analysis	STATA 17 was used for the full statistical and regression analysis. The R statistical software (4.3.1) was additionally used to furnish the statistical analysis and generate the coefficient plots.

For manuscripts utilizing custom algorithms or software that are central to the research but not yet described in published literature, software must be made available to editors and reviewers. We strongly encourage code deposition in a community repository (e.g. GitHub). See the Nature Portfolio guidelines for submitting code & software for further information.

Data

Policy information about availability of data

All manuscripts must include a data availability statement. This statement should provide the following information, where applicable:

- Accession codes, unique identifiers, or web links for publicly available datasets
- A description of any restrictions on data availability
- For clinical datasets or third party data, please ensure that the statement adheres to our policy

Data and code are made publickly available at https://zenodo.org/record/8117848

Research involving human participants, their data, or biological material

Policy information about studies with human participants or human data. See also policy information about sex, gender (identity/presentation), and sexual orientation and race, ethnicity and racism.

Reporting on sex and gender	We do not report sex or gender-based analysis in the manuscript. However, the sex of household-head was used as a covariable in the various regressions. Our findings do not apply to only one sex. Sex was not considered in the study design. Participants (household heads) were randomly selected as explained below. Thus, the proportion of male/female-headed households reflects the population structure. Data were collected using a questionnaire that included a question on the sex of the participant. Inform consent was received from all participants.
Reporting on race, ethnicity, or other socially relevant groupings	The Manuscript does not include any information on race, ethnicity or any other social group.
Population characteristics	The targeted populations for the study are rural groundnut farmers in Ghana, Mali and Nigeria. The target areas are zones of influence of USAID's Feed the Future program. These areas have benefited from several food security and poverty reduction support projects over the past decade.
Recruitment	Participants was recruited based on a multi-stage random sampling. First, villages were selected based on their accessibility and involvement in the groudnut upscaling project. Second, about 30 participants household were randomly selected in each village.
Ethics oversight	The study was approved by the internal research committee of the International Crops Research Institute for the Semi-Arid Tropics

Note that full information on the approval of the study protocol must also be provided in the manuscript.

Field-specific reporting

Please select the one below that is the best fit for your research. If you are not sure, read the appropriate sections before making your selection.

☐ Life sciences ☒ Behavioural & social sciences ☐ Ecological, evolutionary & environmental sciences

For a reference copy of the document with all sections, see nature.com/documents/nr-reporting-summary-flat.pdf

Behavioural & social sciences study design

All studies must disclose on these points even when the disclosure is negative.

Study description	We investigate the relationship between the adoption of climate-resilient groundnut varieties, yields, and smallholder commercialization using panel data from Ghana, Mali, and Nigeria. We find adoption of climate-resilient groundnut varieties to increase yields, production, and production value. Adoption also increases market participation, quantity sold, and sales value. The biggest commercialization gains are observed under the sustained use of these climate-resilient varieties over time. We also show that adoption benefits all households at different quantiles of the conditional distribution of commercialization. However, the gains are more predominant among farmers who produce at smaller scales, suggesting some inclusivity. Our findings are robust to model specifications, and estimation strategies. We conclude that adoption of climate-resilient groundnut varieties can, at least partially, reduce production constraints and promote commercialization.
Research sample	We use a farm household survey that was conducted over three panel years (2017, 2018 and 2019) in Mali, Ghana, and Nigeria. These surveys followed up on the same households enabling us to treat the data as a panel. The data collection followed a multistage procedure where different regions and districts were randomly selected. From these randomly selected districts, 4-6 villages were selected, and households were subsequently randomly selected from these villages. About 30 households were interviewed from these representative villages using survey-based tablets. Data were collected as part of the USAID-funded groundnut upscaling project, implemented by ICRISAT. Then, the survey focused on the intervention area of the project. The multistage sampling procedure describe below was adopted to ensure representative sample.
Sampling strategy	This study follows a multi-stage sampling technique as briefly highlighted above. Groundnut producing districts were randomly selected in the study countries. From these, villages were further selected using the probability proportional to size sampling approach. Finally, households were randomly selected from these villages. In 2017, 900, 1350 and 2500 households were randomly selected from Ghana, Mali and Nigeria respectively. For the second round of surveys, the initial sample sizes were reduced by about 35% due to financial hurdles. In this regard, about 65% of households were again randomly re-sampled into the new sample and interviews were again collected in 2018. Thus, 540, 900 and 1600 households were interviewed in 2018 in Ghana, Mali and Nigeria respectively. These households were again followed in 2019 but the sample size reduced to 506, 840 and 1530 households in Ghana, Mali and Nigeria respectively. This sample was representative. After deleting some missing entries and cleaning the data, we ended up with 2868 households in all three countries giving a total of 8604 observations for the three years. Given that some households attrited (8% in Ghana, 7% in Mali, and 4% in Nigeria) between 2018 and 2019, we performed some attrition probit regressions to be sure that these attrition does not bias our results. Indeed, we find results showing that attrition bias is not an issue in the analysis.
Data collection	The data was collected as part of USAID funded project (2015-2019) on upscaling groundnut production and productivity in Ghana,

Data collection	Mali and Nigeria. A multi-stage sampling technique was used where households cultivating groundnuts were randomly selected and interviewed using questionnaires. Enumerators were used to assist the project team in the data collection process. Data was collected using survey-based tablets. Data collecteion was coordinated by Jourdain Lokossou with the support of well-trained enumerators.
Timing	Data was collected from households in 2017, 2018 and 2019 in Ghana, Mali and Nigeria. Households were sampled in these three years to collect information regarding their production practices. The aim was to identify gaps and needs for upscaling groundnut production.
Data exclusions	No data was excluded from the analysis
Non-participation	About 8%, 7%, and 4% of households dropped out in Ghana, Mali, and Nigeria, respectively due to non availability during the survey period.
Randomization	The data collection followed a multistage procedure where different regions and districts were randomly selected. From these randomly selected districts, villages were selected, and households were subsequently randomly selected from these villages.

Reporting for specific materials, systems and methods

We require information from authors about some types of materials, experimental systems and methods used in many studies. Here, indicate whether each material, system or method listed is relevant to your study. If you are not sure if a list item applies to your research, read the appropriate section before selecting a response.

Materials & experimental systems

n/a	Involved in the study
☒	☐ Antibodies
☒	☐ Eukaryotic cell lines
☒	☐ Palaeontology and archaeology
☒	☐ Animals and other organisms
☒	☐ Clinical data
☒	☐ Dual use research of concern
☒	☐ Plants

Methods

n/a	Involved in the study
☒	☐ ChIP-seq
☒	☐ Flow cytometry
☒	☐ MRI-based neuroimaging
